# High-Purity Phycocyanin Production from Cyanobacteria Using a Biorefinery Approach: Life Cycle Assessment and Comparative Process Benchmarking

**DOI:** 10.3390/microorganisms14061328

**Published:** 2026-06-13

**Authors:** Alejandro Piera, Victoria Morales, Gemma Vicente, Luis Fernando Bautista, Juan José Espada

**Affiliations:** 1 Department of Chemical and Environmental Technology, ESCET, Universidad Rey Juan Carlos, 28933 Móstoles, Spain; alejandro.piera@urjc.es (A.P.); victoria.morales@urjc.es (V.M.); fernando.bautista@urjc.es (L.F.B.); 2 Department of Chemical, Energy and Mechanical Technology, ESCET, Universidad Rey Juan Carlos, 28933 Móstoles, Spain; gemma.vicente@urjc.es; 3 Instituto de Investigación de Tecnologías para la Sostenibilidad, Universidad Rey Juan Carlos, 28933 Móstoles, Spain

**Keywords:** spirulina, high-purity phycocyanin, life cycle assessment, biorefinery

## Abstract

Phycobiliproteins (PBPs) are a family of pigment-proteins renowned for their exceptional light-harvesting, fluorescent, and antioxidant properties. Among cyanobacteria, Spirulina stands out as one of the richest natural sources of PBPs, particularly phycocyanin (PC) and allophycocyanin (APC), yet the large-scale production of analytical-grade PBPs remains hampered by an inherently complex downstream process that relies on multiple purification steps, compromising both yield and scalability. This work presents a streamlined strategy to obtain analytical-grade PC, combining ultrasound-assisted extraction (UAE) with an aqueous ionic liquid (IL) solution and a single hydrophobic interaction chromatography (HIC) step, integrated within a biorefinery framework. The proposed approach yielded analytical-grade PC with a recovery of up to 50.44% and enhanced APC purity up to 10.57-fold. Furthermore, the IL was successfully reused in both extraction and purification steps without compromising yield or purity. The environmental performance of the proposed process was assessed through a cradle-to-gate life cycle assessment (LCA), with system boundaries encompassing the following biorefinery stages: cultivation, harvesting and drying, PC extraction and purification, post-processing, and spent biomass valorization via anaerobic digestion. The LCA identified the main environmental hotspots and guided the proposal of targeted process improvements—particularly HIC salt substitution and increased IL recovery—which reduced environmental impacts by 65.9–89.8% across most categories. The proposed strategy was further benchmarked against two model scenarios for analytical-grade PC production, one conventional and one innovative, revealing its relative advantages and limitations. Overall, this work demonstrates a viable pathway for producing high-purity PC that balances process efficiency with environmental sustainability, supporting the development of greener microalgae-based bioprocesses.

## 1. Introduction

Cyanobacteria, particularly *Limnospira platensis*, commercially known as Spirulina, have emerged as promising biomass feedstocks for biorefinery applications. This is attributed to their high growth rates, CO_2_ fixation capacity, and versatility in product generation, including high-value compounds such as phycocyanin (PC) [1,2,3]. PC is a chromoprotein that works as an accessory photosynthetic pigment to chlorophyll. Its diverse biological activities—including antioxidant, antiviral, anticancer, anti-inflammatory, and neuroprotective effects—have fostered its commercial utilization across multiple sectors, including food, cosmetics, nutraceuticals, pharmaceuticals, biomedicine, and human health [4,5]. The commercial value of PC is significantly influenced by its purity, which is primarily ascertained by the absorbance ratio at 620 nm relative to 280 nm. This A620/A280 ratio serves as the basis for classifying PC purity into distinct grades: food grade (>0.7), cosmetic grade (>1.5), reagent grade (>3.9), and analytical grade (>4.0) [6,7,8,9]. Analytical-grade PC is used in sensitive applications—biomedical, diagnostic, pharmaceutical—where only the highest purity is acceptable. Nevertheless, its production remains constrained by complex, multi-step purification strategies that compromise yield and scalability, translating into elevated market prices [9,10].

The most widely used protocol for analytical-grade PC production involves extraction, often freeze–thaw (FT) or ultrasonication, subsequent precipitation with ammonium sulfate and dialysis, and further purification through ion exchange chromatography (IEC); some methods also incorporate gel filtration or affinity chromatography for enhanced refinement [6,11,12,13]. This sequence is effective for achieving high-purity PC, although it requires multiple, diverse purification stages. This may lead to product losses, long operation time, and complex processes, which can make their industrial scale-up difficult [14,15,16]. When a PC-rich crude extract to be purified contains a significant amount of salt and therefore a considerable ionic strength, IEC may not be the most suitable technique, as it requires either diluting the sample or incorporating intermediate steps, such as precipitation and dialysis, to reduce ionic strength. In this case, hydrophobic interaction chromatography (HIC) becomes particularly attractive [17,18]. HIC exploits high salt concentration to promote hydrophobic interactions between analytes and hydrophobic ligands of the resin. Hydrophobicity is key to achieving strong interactions between proteins and resin ligands. Thus, highly hydrophobic proteins bind more strongly to the ligands than less hydrophobic ones, whose binding with ligands is weak or they are unretained in the column. Elution of adsorbed proteins requires the application of a decreasing salt gradient, which weakens the hydrophobic interactions (protein-ligand), achieving the desorption of the proteins according to their hydrophobicity [17,18]. Compared to other chromatographic techniques, HIC offers greater selectivity for removing undesirable protein variants or misfolded forms, as well as certain types of impurities, such as hydrophobic aggregates. It also offers high resolution to separate proteins with similar charge or size based on their hydrophobic characteristics, and mild purification conditions that preserve the stability and functionality of the purified proteins. However, HIC requires extensive optimization of operating conditions and is not fully compatible with some detergents. Furthermore, the process often requires high salt concentrations to establish sufficient hydrophobic interaction between the protein and the adsorbent, which can imply high operating and disposal costs [17,18,19]. Recently, alternative streamlined routes have been explored, such as aqueous two-phase systems (ATPS) and membrane-based techniques. However, most of these protocols rely on a series of cascade purification steps, and only a few studies have succeeded in achieving the analytical-grade purity required for phycobiliproteins (PBPs) without incorporating at least one chromatographic operation [20,21,22].

From an environmental standpoint, the life cycle assessment (LCA) of analytical-grade PC production highlights significant challenges. The process typically involves several stages—cultivation, harvesting, extraction, and multiple purification steps. Key factors influencing the LCA include the adoption of sustainable production approaches, such as employing industrial wastewater as a growth medium, which can further reduce culture environmental impacts by recycling nutrients and decreasing freshwater demand [23]. Indeed, the integration of industrial side-streams as nutrient sources has been shown to lower the environmental footprint of the cultivation stage while sustaining biomass productivity within commercially relevant ranges [23]. However, current European regulations limit the use of wastewater for cultivating microalgae and cyanobacteria intended for food or cosmetic applications, thereby limiting the potential integration of wastewater-derived nutrients in biorefinery schemes targeting high-value bioproducts [24]. Beyond nutrient-related strategies, higher productivity and resource efficiency are increasingly understood to depend on engineered light distribution and mixing in advanced photobioreactor designs, dense online sensing of culture and environmental parameters, and the incorporation of artificial-intelligence- and machine-learning-based tools capable of predicting and actively optimizing growth conditions in real time [25,26,27]. Harvesting and drying biomass in microalgae and cyanobacteria-based biorefineries are critical processes due to their intensive energy requirements [28], contributing to environmental impacts such as global warming potential [29]. Conventional PC extraction methods typically rely on deionized water, phosphate buffers, or other saline solutions combined with physical cell disruption techniques. While effective, these approaches generate large volumes of saline wastewater requiring appropriate treatment and recycling, entail substantial water consumption during extraction and downstream washing steps, and demand significant energy inputs for processes such as repeated freeze–thaw (FT) cycles [30]. In this context, increasing attention has been devoted to developing alternative extraction strategies that enhance extraction efficiency while concurrently reducing the environmental impact of the process. Such strategies typically rely on less energy-intensive technologies combined with green solvent systems [31]. In this regard, life cycle and related environmental assessments indicate that UAE, combined with suitably chosen green or bio-based solvents, can deliver comparable or higher extraction yields with substantially lower environmental impacts, driven by shorter processing times, lower energy demand, and reduced solvent consumption. However, factors such as solvent production and recovery, downstream processing requirements, and the underlying energy source play a critical role in determining the overall environmental performance: a “green” label alone is not sufficient, and each UAE–solvent combination must be assessed on a case-by-case basis [32,33]. Among the proposed alternatives, ionic liquids (ILs) have been investigated owing to their negligible vapor pressure, high thermal and chemical stability, and highly tunable physicochemical properties—such as polarity and hydrogen-bonding capacity—which can favor enhanced solute solubility and improved selectivity in phycobiliprotein extraction [34,35]. The application of aqueous IL-based systems has been reported to outperform conventional buffer-based extractions in terms of both PC recovery and extract selectivity, reinforcing their potential as a sustainable alternative for phycobiliprotein recovery [36]. The purification stage required to obtain analytical-grade PC represents the most important bottleneck and increases both costs and environmental impacts, particularly when compared with the streamlined processes for lower-purity PC, such as food or cosmetic-grade products [37,38,39,40,41,42]. On the one hand, the preliminary ammonium sulfate precipitation process is water-intensive and requires careful disposal of ammonium sulfate solutions to avoid environmental harm [30,39]. On the other hand, the chromatographic stage is a major hotspot due to its high water and buffer consumption and the finite lifespan of chromatographic resins. Furthermore, column regeneration and cleaning involve additional energy and chemical consumption [43,44]. Regarding alternative purification routes for obtaining analytical-grade PC, ATPS and membrane-based techniques may offer environmental advantages over chromatographic approaches, reducing wastewater generation and energy consumption. However, they seldom achieve analytical-grade purity, and challenges must be addressed, including polymer and salt waste generation, membrane fouling, and the environmental persistence of non-biodegradable polymers. In addition, ATPS often involve high salt concentrations, particularly potassium phosphate, potentially resulting in saline wastewater streams in the absence of appropriate treatment or recycling [20,45]. Membrane techniques also present environmental issues that require careful consideration. In this sense, membrane fouling by proteins and other organic matter requires frequent cleaning, often involving chemicals with potential environmental impacts [46]. Compared to chromatographic columns, membranes have a limited loading capacity and a higher cost per unit of surface area [47]. Additionally, membrane processes consume energy for operation, particularly at larger scales [46].

Overall, the production of analytical-grade PC—whether via conventional IEC or alternative purification methods—poses both technical and environmental challenges. The present work addresses this gap by developing and experimentally validating an integrated process for PC production within a biorefinery framework. UAE with an aqueous IL solution is employed for extraction, after which—in a key methodological departure from prior approaches—the intrinsic high ionic strength of the resulting extract is directly leveraged to enable HIC-based purification without desalting or additional upstream steps. This strategy allows analytical-grade PC purity to be achieved in a single chromatographic step, reducing the number of unit operations and, consequently, simplifying the overall industrial production line. To the best of our knowledge, such an integrated extraction–purification approach has not been reported previously for analytical-grade PC production.

In parallel, LCA is conducted to rigorously evaluate the environmental performance of the proposed process within a conceptual biorefinery framework. Following a cradle-to-gate approach, the system boundaries encompass cultivation, harvesting and drying, PC extraction and purification, PC post-processing, and anaerobic digestion (AD) of the spent biomass for biogas production. Environmental hotspots are systematically identified, and potential improvement strategies are proposed and evaluated to reduce the overall environmental burden of the process. The optimized route is further benchmarked against two contrasting scenarios—a conventional IEC-based scheme and an innovative sequential ATPS-based alternative—providing a robust comparative environmental perspective that supports the design of more efficient and sustainable production strategies for high-purity phycobiliproteins.

## 2. Materials and Methods

### 2.1. Materials

Spirulina was provided as dried powder by AlgaEnergy S.A. (Madrid, Spain); The ionic liquid 1-ethyl-3-methylimidazolium ethyl sulfate ([Emim]^+^[EtSO_4_]^−^), the salts used for buffer preparation (Na_2_HPO_4_, NaH_2_PO_4_, (NH_4_)_2_SO_4_; analytical grade), and the dialysis membrane with a molecular weight cut-off (MWCO) of 14 kDa were obtained from Sigma-Aldrich (St. Louis, MO, USA). Analytical-grade PC and APC standards were obtained from Cymit Química S.L. (Barcelona, Spain). The Butyl-S Sepharose^TM^ 6 Fast Flow HIC resin (BS-FF) was provided by Cytiva Europe GmbH (Freiburg, Germany).

### 2.2. Methods

#### 2.2.1. PC-APC Extraction

Spirulina crude extract was prepared by adapting the extraction procedure reported by Sánchez-Laso et al. (2021, 2023) [48,49]. Dried Spirulina powder (0.18 g) was mixed with 10 mL of an aqueous solution of [Emim]^+^[EtSO_4_]^−^ at 20.86 wt% and stirred for 30 s using an IKA Vortex 3 orbital shaker (IKA-Werke GmbH, Staufen, Germany). The mixture was then subjected to ultrasonic treatment for 20 min at 35 °C with an Elmasonic P system (Elma Schmidbauer GmbH, Singen, Germany) operating at 37 kHz and 656 W. After sonication, the suspension was centrifuged at 5000 rpm for 10 min using an Eppendorf 5910 centrifuge (Eppendorf, Hamburg, Germany) to separate the supernatant from the spent biomass. Finally, the Spirulina crude extract was filtered through a 0.45 μm membrane to remove remaining cell debris and fine impurities. All experiments were performed in triplicate.

#### 2.2.2. PC-APC Purification

The Spirulina crude extract was purified using an Äkta Start system equipped with UNICORN™ Start 1.3 software (both from Cytiva Europe GmbH, Freiburg, Germany), following the method described by Piera et al. (2026) [50]. HIC was carried out using BS-FF resin (bed height: 2.5 cm; radius: 0.35 cm). This resin features a hydrophobic butyl ligand covalently attached to agarose monosaccharide units via glycidyl ether linkages, resulting in an uncharged matrix with highly stable ligand–agarose bonds. The ligand density is 10 μmol·mL^−1^ of resin, and the particle size distribution corresponds to 90 d 50 V.

The resin was preconditioned with 2.5 bed volumes (BV) of buffer solution B (Na_2_HPO_4_/NaH_2_PO_4_; 20 mM; pH 7.0) followed by 7.5 BV of buffer solution A (Na_2_HPO_4_/NaH_2_PO_4_; 20 mM; pH 7.0; 1.25 M (NH_4_)_2_SO_4_). The samples (0.1 mL of filtered Spirulina crude extract) were injected and subjected to an isocratic step at 2.5 M (NH_4_)_2_SO_4_, followed by a linearly decreasing ionic strength gradient of (NH_4_)_2_SO_4_ (from 1.25 to 0 M) at a flow rate of 1.0 mL·min^−1^. Finally, the collected fractions were analysed.

#### 2.2.3. PC and APC Characterization

**UV-Vis spectroscopy**

PC and APC content was determined through spectrophotometric analysis using the equations developed by A. Bennett and L. Bogorad (1973) [51], and the results were expressed as a fraction of the initial cyanobacterial biomass,

(1) $$E_{PC}\left(\frac{\mathrm{mg}\;PC}{g_{biomass}}\right)=\left(\frac{\left({OD}_{615}-0.474\cdot {OD}_{652}\right)}{5.34}\right)\cdot \frac{V_{sample}\left(\mathrm{mL}\right)}{m_{biomass}\left(g\right)}$$

(2) $$E_{APC}\left(\frac{\mathrm{mg}\;APC}{g_{biomass}}\right)=\left(\frac{\left({OD}_{652}-0.208\cdot {OD}_{615}\right)}{5.09}\right)\cdot \frac{V_{sample}\left(\mathrm{mL}\right)}{m_{biomass\;}\left(g\right)}$$

where OD represents the optical density at the wavelength indicated by the subscript (nm).

PC and APC purity (P_PC_, P_APC_) was calculated as a ratio of the maximum absorbance of each one and the whole protein content, as defined by the following equation [52,53]:

(3) $$P=\frac{{OD}_{max}}{{OD}_{280}}$$

The purification efficiency was assessed using the purification factor, which was calculated as the ratio of the purity of PC and APC in the Spirulina crude extract to their respective purity in each purified fraction, as depicted in equation [49]:

(4) $$PF=\frac{P_{PC\;or\;APC\;}\left(purified\;fraction\right)}{P_{PC\;or\;APC}\left(crude\;extract\right)}$$

The measurements were conducted using a spectrophotometer UV-Vis Evolution™ 201/220 (Thermo Fisher Scientific Inc., Waltham, MA, USA).

**Fluorescence spectroscopy**

Fluorescence measurements were performed using an Agilent Cary Eclipse Fluorescence Spectrophotometer (Agilent Technologies, Santa Clara, CA, USA) equipped with Czerny–Turner excitation and emission optics. An 80 Hz Xe pulsed lamp served as the excitation source. Excitation and emission spectra were recorded at 610/623 nm for PC and 610/660 nm for APC, respectively [54,55].

#### 2.2.4. IL Recovery

The IL was recovered through dialysis and rotary evaporation, using the process described by Sánchez-Laso et al. (2021) [48], achieving a recovery yield of 74.8%.

#### 2.2.5. Goal and Scope of the LCA Study

This study evaluated the environmental performance of the experimentally developed process for analytical-grade PC production within a biorefinery framework (Scenario A1), with the dual aim of identifying key environmental hotspots and proposing targeted improvement strategies. An alternative process configuration (Scenario A2) was also assessed, in which ammonium sulfate was replaced by sodium sulfate in the HIC step. Additionally, Scenario A2 incorporated an enhanced IL recovery rate, increased from 74.8 wt% in the baseline scenario to 90 wt%. For comparative purposes, two additional model routes for producing analytical-grade PC were defined based on literature data (see Table S1 in the Supplementary Material). Scenario B represents a conventional process widely used in academic and industrial settings, involving a preliminary purification step followed by IEC [6,56,57,58,59,60], whereas Scenario C corresponds to a more innovative protocol based on a double ATPS, as reported in recent studies [20,21,22]. All scenarios were assessed using a cradle-to-gate approach, with the functional unit defined as the production of 10 g of analytical-grade PC per hour. Based on an annual operation of 8000 h, the resulting pilot plant capacity is 80 kg·year^−1^ of purified PC. The biorefinery was assumed to be in Spain; consequently, background datasets for energy and material production were selected to reflect the Spanish and, where necessary, European context, ensuring full consistency between the foreground and background systems.

#### 2.2.6. System Boundaries

**Figure 1:**
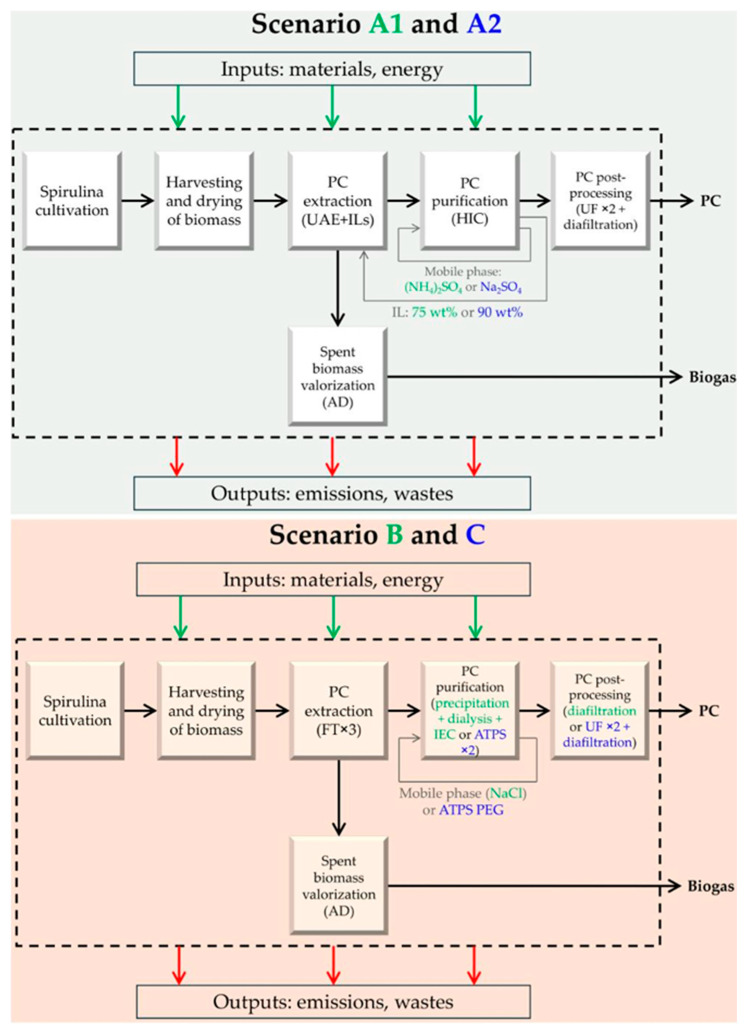
System boundaries of the evaluated scenarios (**A1**, **A2**, **B**, and **C**).

Figure 1 illustrates the system boundaries applied to all evaluated scenarios, which share a common structure encompassing Spirulina cultivation (C), biomass harvesting and drying (H&D), PC post-processing (P-p), and valorization of the spent biomass via anaerobic digestion (V). Scenarios differed exclusively in the PC extraction and purification schemes: Scenarios A1 and A2 employed UAE with a single HIC purification step; Scenario B comprised three FT cycles followed by ammonium sulfate precipitation, dialysis, and IEC; and Scenario C involved three FT cycles succeeded by two consecutive ATPS-based purification stages. For all scenarios, the generation and treatment of wastewater streams produced throughout the process were accounted for within the LCA system boundaries.

#### 2.2.7. Life Cycle Inventory (LCI)

The LCI data for all evaluated scenarios was derived from detailed mass and energy balances obtained through process simulation using SuperPro Designer v9.5 (Intelligen Inc., Scotch Plains, NJ, USA). The complete inventory, including chemical inputs and energy consumption for each scenario, is provided in Table S2 (Supplementary Material), with the corresponding flowsheet diagrams shown in Figures S1–S3. Background system data for material and energy production were sourced from the Ecoinvent 3.9.1 and Sphera LCA databases, with processes selected to match the geographical scope of the foreground system; electricity consumption was modelled using the Spanish national grid mix.

All wastewater streams generated throughout the process consisted of aqueous solutions of inorganic salts and were modelled using the *“Europe without Switzerland: wastewater, average”* dataset (Ecoinvent 3.9.1). The sole exception were the post-purification effluents in Scenarios A1, A2, and C—comprising aqueous solutions of ILs or polyethylene glycol (PEG)—which were classified as hazardous waste and modelled using the “*Treatment and disposal of hazardous waste*” dataset (Ecoinvent 3.9.1). With respect to additional modelling assumptions, the CO_2_ fraction of the biogas produced during anaerobic digestion was not inventoried as a direct atmospheric emission, given that biogas was considered a biofuel output of the process. ILs were modelled as a binary mixture of dimethyl sulfate and imidazole, and their effluents were treated as hazardous material (even though ILs are green solvents) because of possible contamination with hazardous compounds from other stages of the process. Transportation of products and reagents was excluded from the system boundaries.

#### 2.2.8. Modelling Biorefinery Process Stages

**Cultivation (C)**

The cultivation of Spirulina was simulated using solar radiation as the light source within a photobioreactor housed in a greenhouse-type facility. The electricity consumption attributed to the photobioreactor corresponds solely to mechanical energy inputs (agitation and pumping). For a representative location in southern Europe (e.g., southern Spain), the annual average photosynthetically active radiation (PAR) ranges approximately from 20 to 24 mol photons·m^−2^·d^−1^ outdoors [61,62]; accounting for the typical light transmittance of greenhouse covering materials (60–80% for glass or polycarbonate panels), the estimated PAR reaching the culture surface is approximately 14–19 mol photons·m^−2^·d^−1^ [63,64]. A simplified Zarrouk medium was employed, consisting of sodium bicarbonate (16.8 g·L^−1^) and dissolved CO_2_ as inorganic carbon sources, together with sodium nitrate (0.25 g·L^−1^) as the nitrogen source. It should be noted that the nitrogen input value reported in the life cycle inventory does not reflect a static initial batch concentration, but rather the net nitrogen flux entering the system derived from the mass balance at steady state, accounting for both fresh medium additions and recirculation streams. The culture was maintained at an operational temperature of 30 °C and a pH of 9.0–11 [65], consistent with the usual growth conditions reported for *Limnospira platensis* in greenhouse photobioreactor systems [66]. The culture was modelled in a continuous photobioreactor operating at steady state, with a hydraulic retention time of 8 days. Cell growth was described using Monod’s kinetic model with a maximum specific growth rate (μ_max_) of 0.018 h^−1^ [67,68]. The resulting biomass concentration was 0.48 g·L^−1^, which falls at the lower end of the range typically reported for *Limnospira platensis* in pilot- and pre-commercial scale systems under standard, non-optimised conditions (0.4–0.9 g·L^−1^) [69]. This value was deliberately adopted as a conservative assumption, consistent with the overall methodological approach of this study, in order to avoid overstating the environmental performance of the cultivation stage.

**Harvesting and drying of biomass (H&D)**

Biomass was harvested by a sequential decantation centrifugation process, widely used in industrial microalgal processing [70]. Considering that typical Spirulina trichomes have a diameter ranging from 3 to 12 μm and a variable length starting at approximately 50 μm [71,72]. A particle size of 50 μm was assumed for the centrifugation process, resulting in 98 wt% biomass recovery (2 wt% loss in the supernatant). The resulting wet biomass was subsequently processed by lyophilization, which comprised an initial freezing stage at −50 °C at a cooling rate of 0.5 °C·min^−1^, followed by a primary drying stage up to a final temperature of 25 °C at a sublimation rate of 1.0 mm·h^−1^ [73].

**PC extraction (E) and purification (P)**

The extraction and purification stages for Scenarios A1 and A2 were modelled following the experimental procedures described in Section 2.2.1. PC-APC extraction and Section 2.2.2. PC-APC purification.

Regarding PC extraction, at laboratory scale, freeze-dried Spirulina biomass was processed using an ultrasonic bath (656 W, 6.9 L total volume, 20 min), with a specific energy consumption of 0.0317 kWh·L^−1^ (114.1 J·mL^−1^). At pilot scale, however, extraction was simulated using a probe-based ultrasonication system coupled to a flow cell—a configuration better suited to the throughput volumes involved and known to achieve higher energy transfer efficiency than bath sonicators, which incur significant losses to the surrounding medium and reactor walls. Accordingly, the energy input experimentally determined for the bath setup was applied to the pilot-scale simulation as a conservative upper-bound estimate, scaled proportionally to the solvent volumes reported in the LCI data (Table S2). The resulting PC-APC-rich crude extract was then separated from the spent biomass by decanter centrifugation. In Scenarios B and C, PC-APC extraction was simulated using three consecutive FT cycles, employing acetate buffer (Scenario B) or phosphate buffer (Scenario C) as the extraction solvent, keeping the biomass-to-solvent ratio identical to that used in Scenarios A1 and A2 as reported in the literature [48,58,60].

Concerning purification, the chromatographic step in Scenarios A1 and A2 was scaled up from a fixed-bed adsorption column packed with BS-FF resin. Buffer supply to the column was simulated using three peristaltic pumps: two supplying buffer solutions A and B (as described in the Materials and Methods Section), and one supplying deionized water for resin regeneration. Scale-up was performed maintaining constant linear velocity and bed height, thereby preserving the analyte–stationary phase contact time and ensuring separation performance equivalent to that of the laboratory-scale system [74,75]. The increased flow rates required at the pilot scale were accommodated by proportionally enlarging the column cross-sectional area. Mobile phase reuse rates of 99% and 50% were assumed for the conditioning and stabilization steps, and for the pre-elution and regeneration steps, respectively (Table S3). In Scenario B, preliminary purification was modelled as a two-step ammonium sulfate precipitation at 25% and 60% saturation, followed by resuspension of the precipitate in acetate buffer and subsequent dialysis, yielding a partially purified PC fraction. Based on literature data, a purity of 2.75 (A_620_/A_280_) and a PC recovery yield of 80.1 wt% were assumed for this step (see Table S4) [60]. This partially purified fraction was then subjected to chromatographic purification using a weak anion-exchange IEC column, scaled up following an analogous procedure to that applied to the HIC column in Scenarios A1 and A2. Column parameters (bed volume: 8 mL, radius: 0.8 cm, sample load: 1.25 BV) were sourced from the literature compiled in Table S1 (Supplementary Material), while the corresponding mobile-phase volumes and recycled salt amounts are reported in Table S3 (Supplementary Material). In Scenario C, purification was carried out through two consecutive ATPS stages, with phase separation achieved by decanter centrifugation. Process parameters and assumed performance values—a PC recovery yield of 80% and a purity ratio of 3.92 (A_620_/A_280_) after the second ATPS stage—were sourced from the literature compiled in Tables S2 and S4 (Supplementary Material) [20,21,22].

**Post-processing (P-p)**

Following chromatographic purification, the recovered PC fraction typically presents reduced product concentration and elevated salt content, necessitating additional concentration and desalting steps to meet the purity specifications required for commercial analytical-grade applications [76,77]. In Scenarios A1 and A2, this was addressed through a sequential post-processing train in which two consecutive UF units were used to concentrate the PC fraction, followed by a diafiltration step to remove the excess salt carried over from the HIC elution buffer. Each UF unit was modelled with a concentration factor of 5, a filtrate flux of 20 L·m^−2^·h^−1^, and membrane areas of 9.85 and 5.21 m^2^, respectively. This post-processing sequence yielded analytical-grade PC at a production rate of 10 g·h^−1^ and a final concentration of 1 g·L^−1^ in 10 mM sodium phosphate buffer. In Scenario B, a single diafiltration step was simulated to remove the excess salt remaining after IEC elution. In Scenario C, a UF step was first applied to concentrate the PC fraction and reach the analytical-grade purity threshold (A_620_/A_280_ > 4), as the purity attained after the second ATPS stage was slightly below this value, followed by two consecutive diafiltration steps to remove residual salts and polymer carried over from the ATPS stages. Additional details for both scenarios are provided in Table S4 (Supplementary Material).

**Spent biomass valorization (V)**

The spent biomass recovered from the extraction stage was subsequently valorized by anaerobic digestion (AD). The operating conditions—a substrate-to-inoculum ratio of 1:2 and a substrate-to-co-substrate ratio of 4:1 (both on a volatile solids, VS, basis)—were established from preliminary batch AD studies conducted by the research group. In those experiments, spent biomass moisture content was first adjusted from 90 to approximately 80 wt% by centrifugation as a preconditioning step, and anaerobic sludge from a municipal wastewater treatment plant served as inoculum and glycerol—a biodiesel by-product—as co-substrate, under mesophilic conditions (37 °C). Consistent with this experimental protocol, both the preconditioning step—simulated as decanter centrifugation at pilot scale—and the AD process were incorporated into the pilot-scale simulation. The digester was modelled using a stoichiometric conversion based on the experimentally determined elemental composition of the spent biomass: 1.13 C_5_H_8_NO_2_ + 3 H_2_O → NH_3_ + 2.5 CO_2_ + 3.5 CH_4_, and sized at 26.82 m^3^ with a hydraulic retention time (HRT) of 30 days and a working-to-vessel volume ratio of 90%. The process yielded two output streams: biogas (66.2 wt% CH_4_, 33.8 wt% CO_2_) and digestate.

#### 2.2.9. Methodology of Environmental Impact Assessment

Environmental impacts were assessed using a midpoint-oriented approach. The selected impact categories included abiotic depletion potential (ADP, expressed in kg Sb-eq.), Acidification Potential (AP, in kg SO_2_-eq.) greenhouse gas emissions quantified as 100-year Global Warming Potential (GWP, in kg CO_2_-eq), eutrophication potential (EP, in kg PO_4_^3−^-eq), as well as freshwater aquatic ecotoxicity potential (FAETP), human toxicity potential (HTP), and terrestrial ecotoxicity potential (TETP), the three expressed in kg 1,4-dichlorobenzene equivalents (1,4-DCB-eq); and the photochemical ozone creation potential (POCP, in kg ethene-eq). All selected impact categories were quantified in accordance with the CML 2001 methodology. In addition, cumulative energy demand (CED, in MJ) was calculated to account for direct and indirect primary energy consumption across the life cycle, considering both fossil and renewable energy sources [78].

#### 2.2.10. Statistical Analysis

Statistical analyses were carried out to evaluate differences among purification levels (L1–L5) for each phycobiliprotein, and between fresh and reused IL on extraction yield and extract purity. Prior to hypothesis testing, the assumptions of normality and homogeneity of variances were verified by applying the Shapiro–Wilk test to model residuals and Levene’s test, respectively. Since all datasets satisfied both assumptions, differences among methods were assessed using one-way analysis of variance (ANOVA). Whenever significant effects were detected (*p*-value < 0.05), pairwise comparisons were performed using Tukey’s honestly significant difference (HSD) test. Comparisons between fresh and reused solvents were conducted using independent-samples Student’s t-tests. To account for multiple pairwise comparisons, *p*-values were adjusted using Holm’s procedure, and significance was established at α = 0.05. All extraction experiments were carried out in triplicate. Data are presented as mean ± standard deviation (SD). All analyses were performed using Statgraphics Centurion v17.2.07 (Statgraphics Technologies, Inc., The Plains, VA, USA).

## 3. Results

### 3.1. PC-APC Extraction

The PC and APC content of the Spirulina crude extract is summarized in Table 1. Extraction yields (E_PC_, E_APC_) remained consistent, with no significant differences between fresh and reused ILs (*p*-value > 0.05). However, a decrease in product purity was observed when reused ILs were employed (*p*-value < 0.05), indicating a slight, but significant reduction in purification selectivity.

### 3.2. PC-APC Purification

**Figure 2:**
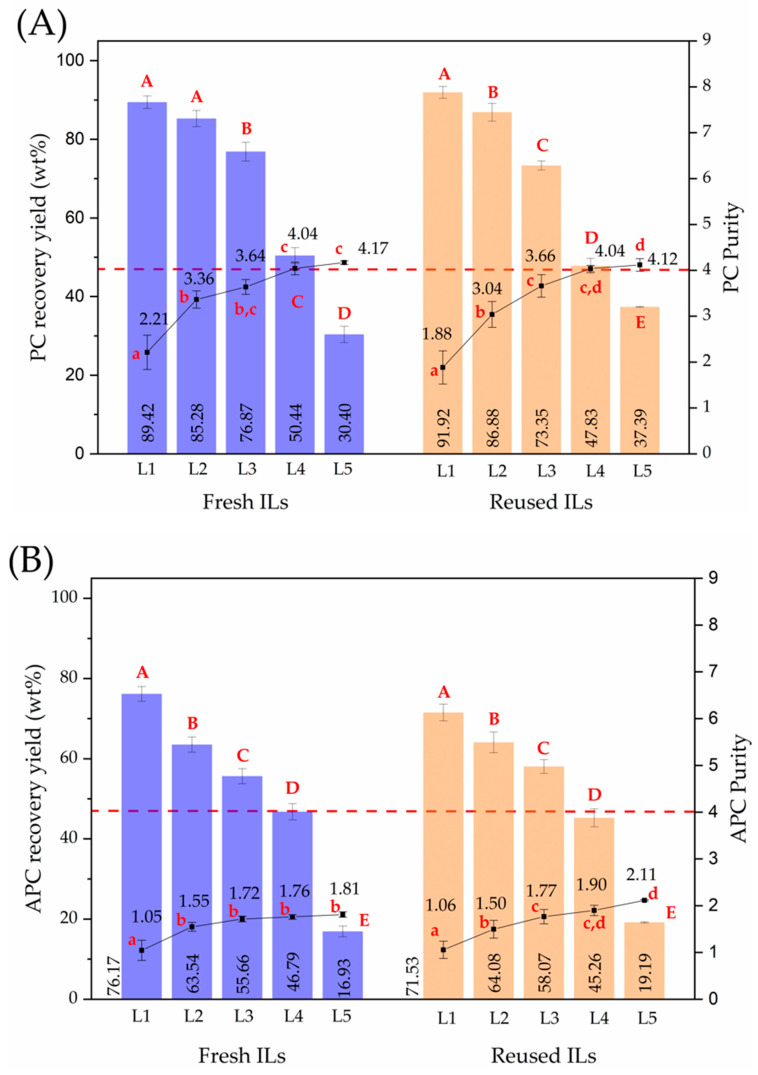
Recovery yield (bars) and purity (dots) of PC (**A**) and APC (**B**) achieved by HIC using BS-FF resin for the purification of Spirulina crude extract with fresh (blue) and reused (orange) ILs. The dashed line indicates the analytical-grade purity threshold (≥4.0). Different letters indicate statistically significant differences among groups based on post hoc multiple comparison tests with adjustment for multiple comparisons (*p*-value < 0.05). Capital letters refer to performance and lowercase letters to purity.

**Figure 3:**
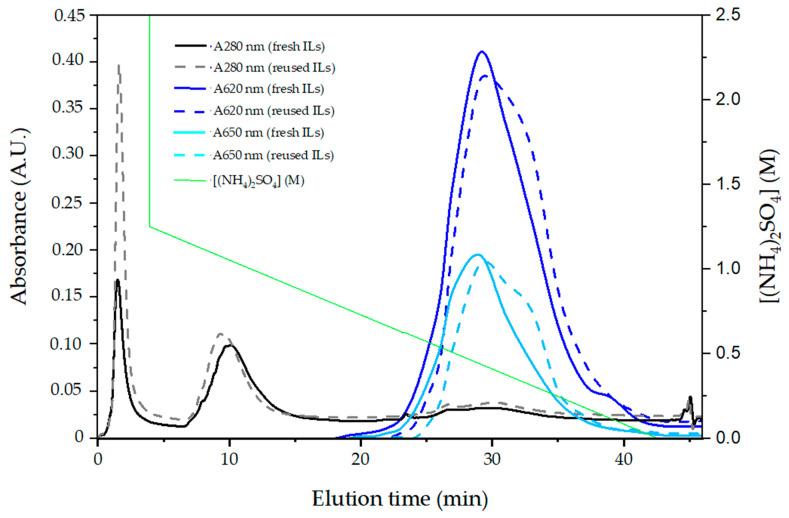
Chromatogram of the HIC purification of Spirulina crude extract using BS-FF resin.

The Spirulina crude extract was purified by single-step HIC in three independent replicates. PC and APC purity, recovery yield, and purification factor (PF) were determined for the collected fractions, with total amounts calculated by summing all fractions in which PC or APC was quantified. Five pooling levels (L1–L5) were defined based on the trade-off between recovery and purity, ranging from the complete set of collected fractions (L1) to the single fraction at the elution peak maximum (L5); for each level, the bar represents the cumulative recovery yield of the pooled fractions, while the dot indicates their mean purity (Figure 2). As expected, progressively restricting the number of pooled fractions increased PC and APC purity at the expense of recovery yield. The reported analytical-grade PC recovery corresponded to the highest recovery yield obtained while satisfying the analytical-grade purity criterion (A620/A280 ≥ 4.0), which was achieved at pooling level L4. This fractionation strategy enabled precise quantification of the recovered PC and APC across defined purity levels, as shown in Figure 2 and Table S5 (Supplementary Material). The chromatographic elution profile, including the elution peak maximum corresponding to L5, is shown in Figure 3.

As shown in Figure 2A, when fresh ILs were used, 50.44 wt% of the total PC in the crude extract was recovered as analytical-grade PC, representing a 7.09-fold increase in purity. On the other hand, using reused ILs yielded a similar outcome, with 47.83% of analytical-grade PC recovered and a 10.05-fold improvement in purity. A comparable trend was observed for APC purification (Figure 2B). Although analytical-grade purity was not achieved, APC purity increased relative to the crude extract, with improvements of up to 6.71-fold with fresh ILs and 10.57-fold with reused ILs.

### 3.3. PC and APC Characterization

The chromatographic and spectroscopic characterization presented in this section was primarily intended to compare the performance of fresh and reused ILs and to experimentally validate the extraction–purification data subsequently used for process simulation and environmental assessment.

**UV-Vis spectroscopy**

**Figure 4:**
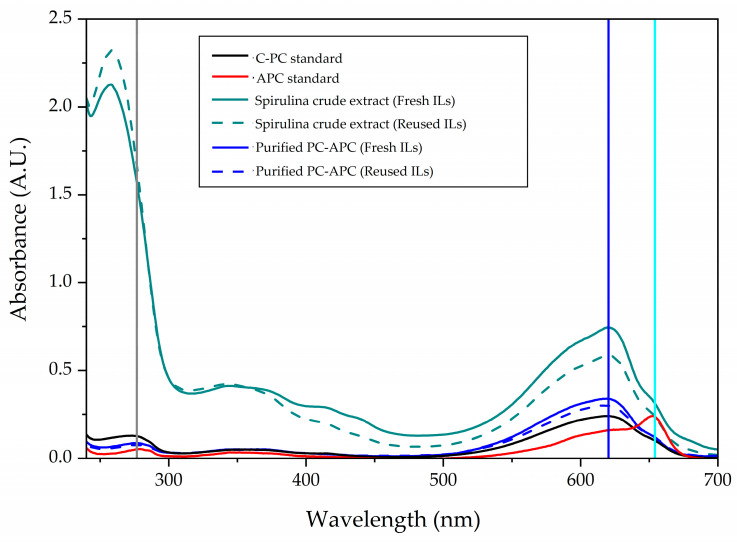
Absorbance spectrum of the Spirulina crude extract, purified PC–APC, C-PC standard, and APC standard. Grey, dark blue, and light blue vertical lines indicate the characteristic maximum absorption wavelengths of proteins, PC, and APC, respectively.

The absorption spectrum obtained for Spirulina crude extract (Figure 4) showed a peak at 260 nm, indicating the presence of proteins and nucleic acids, along with a distinct peak at 620 nm corresponding to PC. A less pronounced peak at 650 nm was attributed to APC. The higher absorbance at 280 nm relative to 620 nm further indicated the presence of additional proteins besides PC [79].

**Fluorescence assay**

**Figure 5:**
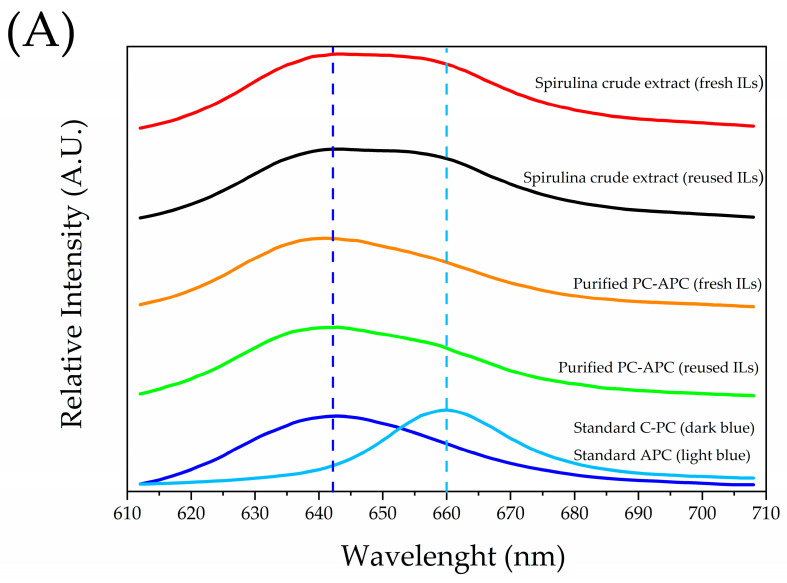

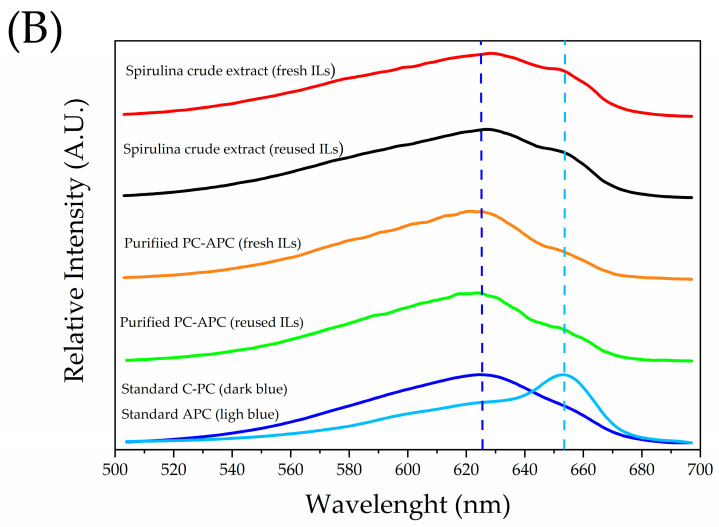
(**A**) Fluorescence emission spectrum of Spirulina crude extracts (fresh and reused ILs), purified PC-APC (fresh and reused ILs), and C-PC and APC standards; (**B**) fluorescence excitation spectrum (λ_ex_ = 610 nm) of Spirulina crude extracts (fresh and reused ILs), purified PC-APC (fresh and reused ILs), and C-PC and APC standards.

Fluorescence spectroscopy was employed to assess potential conformational changes in PC and APC induced by the purification process. The fluorescence emission spectrum of the Spirulina crude extract and the purified PC–APC obtained with both fresh and reused IL showed a characteristic peak at 643 nm and a weaker secondary peak at 660 nm, corresponding to PC and APC fluorescence [55,80] (Figure 5A). The excitation spectrum similarly matched the absorption peaks at 620 nm and 650 nm, reflecting the distinct spectral signatures of PC and APC, respectively [54] (Figure 5B).

### 3.4. Life Cycle Assessment (LCA)

#### 3.4.1. Scenarios A1 and A2

**Figure 6:**
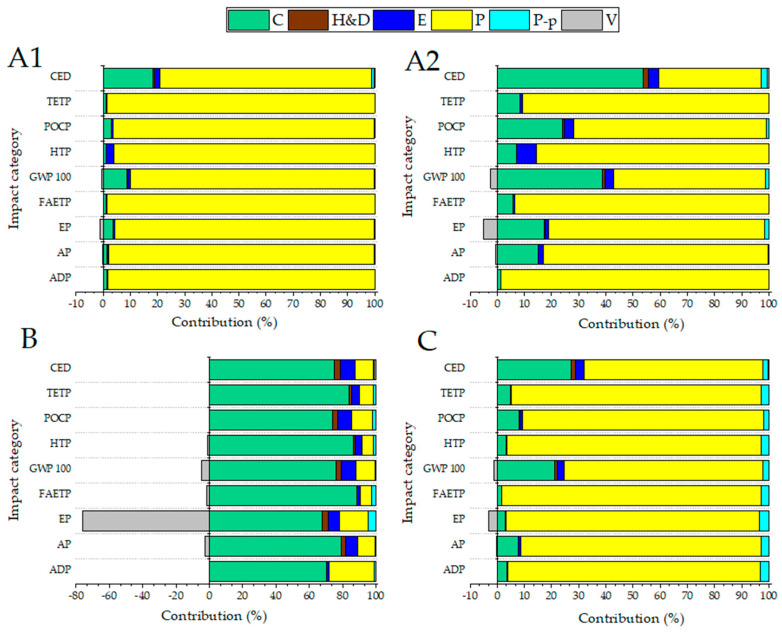
Normalized contributions of the different biorefinery stages to the environmental impacts categories of the studied cases (**A1**–**C**). Cultivation (C); harvesting and drying (H&D); extraction (E); purification (P); post-processing (P-p); and spent biomass valorization (V).

Absolute characterization results for all impact categories in Scenarios A1 and A2, disaggregated by biorefinery stage, are reported in Table 2. The corresponding relative contributions of each stage, expressed as a percentage, are illustrated in Figure 6, enabling the identification of the main environmental hotspots across the system. In both scenarios, the purification stage (P) was identified as the primary environmental hotspot; however, this effect was markedly more pronounced in Scenario A1, accounting for 78.1–98.6% of total impacts across most categories. In this Scenario, the cultivation stage (C) emerged as the second most relevant contributor, although at a substantially lower magnitude, ranging from 1.0% to 8.8% across all impact categories, except cumulative energy demand (CED), where it accounted for 18.3% of the total biorefinery impact. This was followed by the PC extraction stage (E), the harvesting and drying stage (H&D), and the post-processing stage (P-p), whose contribution remained marginal (0.0–0.8%). Finally, the valorization of spent biomass through AD showed the most favorable environmental performance, contributing between −1.1% and 0.2% to the total impacts. In some categories, negative values were observed due to the use of residual streams (anaerobic sludge and glycerol) as feedstock, as shown in Figure 6.

**Figure 7:**
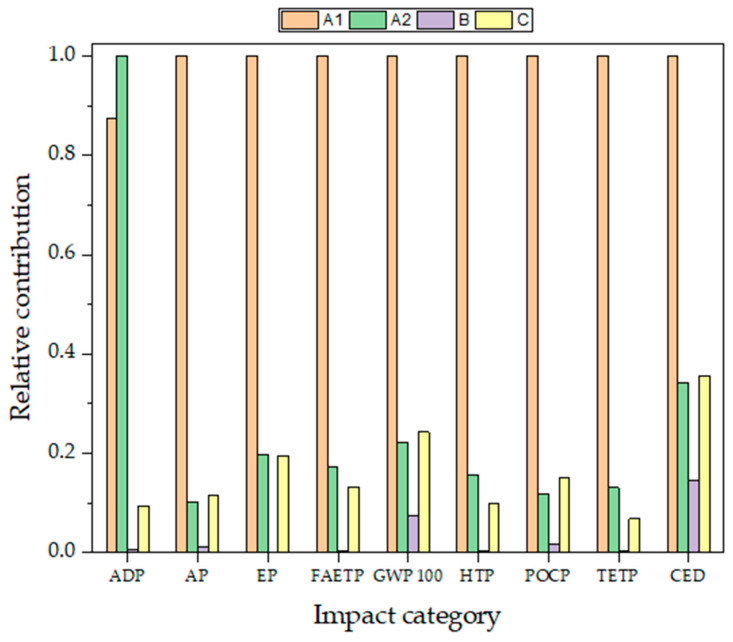
Normalized comparison of LCA results for the studied cases: Scenario A1 (orange); Scenario A2 (green); Scenario B (purple); and Scenario C (yellow).

In Scenario A2, the implemented improvements—including substituting ammonium sulfate with sodium sulfate during HIC and increasing IL recovery to 90 wt%—led to an overall reduction in the absolute environmental impacts of the biorefinery (Table 2). Despite this overall improvement, the purification stage remained the dominant environmental hotspot across most impact categories, accounting for 57.2–98.7% of the total system burden. However, purification was no longer the most influential stage in the cumulative energy demand (CED) category, accounting for only 37.6% of the total impact. In this category, the cultivation stage became the main contributor, accounting for 53.7% of the total CED. As in Scenario A1, cultivation ranked as the second most relevant contributor, with stage contributions ranging from 1.2% to 53.7% across impact categories, and showing a particularly notable influence on global warming potential (GWP 100, 39.5%) and photochemical ozone creation potential (POCP, 24.1%). The remaining stages exhibited comparatively minor contributions: harvesting and drying (0.0–1.9%), extraction (0.1–7.5%), post-processing (0.0–1.7%), and spent biomass valorization (−5.1–0.7%), as illustrated in Figure 6. A normalised comparison between scenarios is provided in Figure 7.

#### 3.4.2. Scenarios B and C

Characterization results for Scenarios B and C, disaggregated by biorefinery stage, are reported in Table 2, while the corresponding relative contributions are illustrated in Figure 6. In Scenario B, the comparatively low contribution of the purification stage (6.9–27.2%) shifted the relative prominence of other stages within the overall life-cycle burden. Consequently, cultivation emerged as the dominant environmental hotspot, accounting for 67.8–88.5% of total impacts, while spent biomass valorization via AD yielded pronounced negative contributions in several categories, reaching −76.0% in eutrophication potential (EP), attributable to the environmental credits associated with the use of waste-derived feedstocks—primarily anaerobic sludge and, to a lesser extent, glycerol—as process inputs. The remaining stages exhibited minor contributions throughout: PC extraction (1.4–8.7%), harvesting and drying (0.2–3.5%), and PC post-processing (0.4–4.8%). In Scenario C, as observed in Scenario A2, the purification stage remained the dominant environmental hotspot across most impact categories, accounting for 65.8–95.3% of the total impact value. Cultivation ranked as the second most relevant contributor, albeit at a lower magnitude (1.6–8.0%), with the highest influence observed in cumulative energy demand (CED, 27.3%) and global warming potential (GWP 100, 21.2%). The remaining stages exhibited minor contributions throughout: PC post-processing (1.9–3.6%), PC extraction (0.1–3.6%), and harvesting and drying (0–1.3%). Spent biomass valorization via AD showed the most favorable environmental performance, yielding contributions ranging from −3.1% to 0.4% across all evaluated categories.

**Table 2:** Overall impacts of Scenarios A1, A2, B, and C referred to the production of 10 g·h^−1^ of analytical-grade PC: cultivation (C); harvesting and drying (H&D); extraction (E); purification (P); post-processing (P-p); and spent biomass valorization (V).

	Impact Category	Unit	C	H&D	E	P	P-p	V	Total
Scenario A1	ADP	kg Sb eq.	1.36 × 10^−4^	3.93 × 10^−7^	2.31 × 10^−5^	9.74 × 10^−3^	1.09 × 10^−6^	−3.74 × 10^−8^	9.90 × 10^−3^
	AP	kg SO_2_ eq.	5.48 × 10^−2^	9.71 × 10^−4^	1.41 × 10^−2^	3.52	1.19 × 10^−3^	−2.07 × 10^−3^	3.59
	EP	kg PO_4_^3−^ eq.	3.50 × 10^−2^	3.76 × 10^−4^	7.69 × 10^−3^	9.32 × 10^−1^	3.00 × 10^−3^	−1.04 × 10^−2^	9.68 × 10^−1^
	FAETP	kg DCB eq.	3.78	3.17 × 10^−3^	1.46	3.80 × 10^2^	1.18 × 10^−2^	−2.69 × 10^−2^	3.86 × 10^2^
	GWP 100	kg CO_2_ eq.	2.57 × 10^1^	7.85 × 10^−1^	3.36	2.64 × 10^2^	9.41 × 10^−1^	−1.61	2.93 × 10^2^
	HTP	kg DCB eq.	9.79	4.73 × 10^−2^	2.63 × 10^1^	8.58 × 10^2^	6.14 × 10^−2^	−5.11 × 10^−2^	8.94 × 10^2^
	POCP	kg Ethene eq.	5.96 × 10^−3^	1.57 × 10^−4^	1.72 × 10^−3^	2.00 × 10^−1^	1.85 × 10^−4^	1.34 × 10^−5^	2.08 × 10^−1^
	TETP	kg DCB eq.	8.17 × 10^−2^	5.37 × 10^−4^	2.06 × 10^−2^	7.33	9.83 × 10^−4^	−1.70 × 10^−4^	7.43
	CED	MJ	1.11 × 10^3^	3.98 × 10^1^	1.16 × 10^2^	4.72 × 10^3^	4.60 × 10^1^	1.41 × 10^1^	6.04 × 10^3^
Scenario A2	ADP	kg Sb eq.	1.36 × 10^−4^	3.93 × 10^−7^	9.50 × 10^−6^	1.12 × 10^−2^	1.09 × 10^−6^	−3.74 × 10^−8^	1.13 × 10^−2^
	AP	kg SO_2_ eq.	5.48 × 10^−2^	9.71 × 10^−4^	6.42 × 10^−3^	3.04 × 10^−1^	1.19 × 10^−3^	−2.07 × 10^−3^	3.65 × 10^−1^
	EP	kg PO_4_^3−^ eq.	3.50 × 10^−2^	3.76 × 10^−4^	3.18 × 10^−3^	1.61 × 10^−1^	3.00 × 10^−3^	−1.04 × 10^−2^	1.92 × 10^−1^
	FAETP	kg DCB eq.	3.78	3.17 × 10^−3^	5.83 × 10^−1^	6.25 × 10^1^	1.18 × 10^−2^	−2.69 × 10^−2^	6.68 × 10^1^
	GWP 100	kg CO_2_ eq.	2.57 × 10^1^	7.85 × 10^−1^	1.98	3.71 × 10^1^	9.41 × 10^−1^	−1.61	6.49 × 10^1^
	HTP	kg DCB eq.	9.79	4.73 × 10^−2^	1.05 × 10^1^	1.20 × 10^2^	6.14 × 10^−2^	−5.11 × 10^−2^	1.40 × 10^2^
	POCP	kg Ethene eq.	5.96 × 10^−3^	1.57 × 10^−4^	8.11 × 10^−4^	1.76 × 10^−2^	1.85 × 10^−4^	1.34 × 10^−5^	2.47 × 10^−2^
	TETP	kg DCB eq.	8.17 × 10^−2^	5.37 × 10^−4^	8.63 × 10^−3^	8.90 × 10^−1^	9.83 × 10^−4^	−1.70 × 10^−4^	9.82 × 10^−1^
	CED	MJ	1.11 × 10^3^	3.98 × 10^1^	7.92 × 10^1^	7.74 × 10^2^	4.60 × 10^1^	1.41 × 10^1^	2.06 × 10^3^
Scenario B	ADP	kg Sb eq.	4.38 × 10^−5^	1.97 × 10^−7^	8.58 × 10^−7^	1.69 × 10^−5^	5.81 × 10^−7^	−5.09 × 10^−8^	6.23 × 10^−5^
	AP	kg SO_2_ eq.	3.57 × 10^−2^	1.29 × 10^−3^	3.29 × 10^−3^	4.55 × 10^−3^	2.64 × 10^−4^	−1.23 × 10^−3^	4.38 × 10^−2^
	EP	kg PO_4_^3−^ eq.	6.16 × 10^−3^	3.16 × 10^−4^	6.25 × 10^−4^	1.54 × 10^−3^	4.35 × 10^−4^	−6.90 × 10^−3^	2.17 × 10^−3^
	FAETP	kg DCB eq.	9.23 × 10^−1^	2.43 × 10^−3^	2.25 × 10^−2^	6.73 × 10^−2^	2.74 × 10^−2^	−1.79 × 10^−2^	1.02
	GWP 100	kg CO_2_ eq.	1.73 × 10^1^	7.90 × 10^−1^	1.99	2.58	1.11 × 10^−1^	−1.03	2.17 × 10^1^
	HTP	kg DCB eq.	3.09	4.69 × 10^−2^	1.45 × 10^−1^	2.46 × 10^−1^	4.98 × 10^−2^	−3.14 × 10^−2^	3.55
	POCP	kg Ethene eq.	2.87 × 10^−3^	1.16 × 10^−4^	3.21 × 10^−4^	5.01 × 10^−4^	6.29 × 10^−5^	4.89 × 10^−6^	3.87 × 10^−3^
	TETP	kg DCB eq.	2.81 × 10^−2^	5.22 × 10^−4^	1.60 × 10^−3^	2.83 × 10^−3^	5.10 × 10^−4^	−6.12 × 10^−5^	3.35 × 10^−2^
	CED	MJ	6.64 × 10^2^	3.07 × 10^1^	7.70 × 10^1^	9.78 × 10^1^	3.95	8.99	8.83 × 10^2^
Scenario C	ADP	kg Sb eq.	3.87 × 10^−5^	1.85 × 10^−7^	1.96 × 10^−6^	9.82 × 10^−4^	3.31 × 10^−5^	−5.26 × 10^−8^	1.06 × 10^−3^
	AP	kg SO_2_ eq.	3.15 × 10^−2^	1.21 × 10^−3^	3.39 × 10^−3^	3.70 × 10^−1^	1.16 × 10^−2^	−1.09 × 10^−3^	4.16 × 10^−1^
	EP	kg PO_4_^3−^ eq.	5.44 × 10^−3^	2.89 × 10^−4^	6.28 × 10^−4^	1.82 × 10^−1^	6.97 × 10^−3^	−6.09 × 10^−3^	1.89 × 10^−1^
	FAETP	kg DCB eq.	8.15 × 10^−1^	2.26 × 10^−3^	7.27 × 10^−2^	4.88 × 10^1^	1.49	−1.58 × 10^−2^	5.11 × 10^1^
	GWP 100	kg CO_2_ eq.	1.53 × 10^1^	7.42 × 10^−1^	1.83	5.26 × 10^1^	1.64	−9.07 × 10^−1^	7.12 × 10^1^
	HTP	kg DCB eq.	2.73	4.40 × 10^−2^	2.17 × 10^−1^	8.25 × 10^1^	2.48	−2.78 × 10^−2^	8.79 × 10^1^
	POCP	kg Ethene eq.	2.53 × 10^−3^	1.09 × 10^−4^	2.85 × 10^−4^	2.78 × 10^−2^	6.01 × 10^−4^	3.96 × 10^−6^	3.13 × 10^−2^
	TETP	kg DCB eq.	2.48 × 10^−2^	4.90 × 10^−4^	1.81 × 10^−3^	4.67 × 10^−1^	1.42 × 10^−2^	−6.27 × 10^−5^	5.08 × 10^−1^
	CED	MJ	5.86 × 10^2^	2.88 × 10^1^	7.01 × 10^1^	1.41 × 10^3^	4.00 × 10^1^	7.91	2.15 × 10^3^

#### 3.4.3. Comparison of Scenarios

Figure 7 presents the environmental performance of all scenarios relative to Scenario A1, which exhibited the highest overall impacts across the biorefinery configurations assessed. The improvements implemented in Scenario A2 resulted in substantial reductions across all impact categories (65.9–89.8%), except for abiotic depletion potential (ADP), which increased by 12.4%—an aspect further discussed in Section 4. Scenario B consistently showed the lowest relative impacts, representing the most environmentally favorable configuration overall. Nevertheless, Scenario A2 demonstrated competitive environmental performance relative to Scenario C, with comparable or lower impacts in acidification potential (AP), photochemical ozone creation potential (POCP), terrestrial ecotoxicity potential (TETP), and global warming potential (GWP 100).

## 4. Discussion

### 4.1. PC-APC Extraction and Purification

Continuing the line of previous research conducted by our research group, the PC extraction protocol developed by Sánchez-Laso et al. (2021, 2023) was employed [48,49]. The protocol relies on [EMIM]^+^[EtSO_4_]^−^ as the extraction solvent, given its demonstrated compatibility with proteins: relative to other imidazolium ILs, it causes less structural perturbation [81], prevents aggregation [82], and allows conformational recovery upon removal [83], as supported by the preserved spectroscopic integrity markers and fluorescence profiles obtained across all tested conditions (Figure 4 and Figure 5).

IL reuse had no substantial effect on extraction yield, with only minor variations observed, as already mentioned previously in this work [48]. However, a more pronounced decrease in PC and APC purity was observed upon IL reuse, suggesting a reduction in purification selectivity. This may be attributed to the accumulation of residual low-molecular-weight proteins and amino acids—retained below the dialysis membrane cutoff—that are reincorporated into subsequent extraction cycles during IL recovery. This phenomenon is reflected in the increased absorbance at 280 nm observed when recycled ILs were used as the extraction solvent (Figure 4).

With respect to purification, the process developed by Piera et al. (2026) for isolating phycoerythrin from red algae was adapted and applied to the purification of PC and APC from the cyanobacterium Spirulina [50]. In this work, the method proved highly efficient for both PC and APC, achieving purification factors of up to 10.05-fold and 10.57-fold, respectively. In addition, the purified fractions obtained after HIC exhibited absorption spectra closely resembling those of the commercial PC standard. The absorbance peaks related to non-target compounds present in the Spirulina crude extract were effectively eliminated, confirming the successful removal of residual proteins and other impurities—such as nucleic acids (absorption at 260 nm) and UV-absorbing metabolites in the 300–360 nm region, which are typically involved in photoprotection in cyanobacteria such as mycosporine-like amino acids [79]. Fluorescence was also assessed, and the spectra of the purified fractions closely matched those of the commercial C-PC and APC standards, confirming the preservation of native protein structure and chromophore stability throughout extraction and purification [84,85].

The literature on PC purification HIC is limited; however, a few studies have explored this approach. Abalde et al. (1999) directly purified crude extracts of *Synechococcus* sp., which are naturally rich in PC, using three HIC resins with different ligands—butyl, octyl, and phenyl [86]. The highest performance was obtained with the butyl resin, yielding a PC purity of 3.2 and a recovery of 83.4%, values that remain far below the analytical-grade purity achieved in the present study. In contrast, phenyl and octyl-agarose were reported as unsuitable under the evaluated conditions, as PC adsorbed too strongly to these resins, leading to very low recovery yields [86]. Soni et al. (2008) demonstrated the feasibility of purifying PC using a hydrophobic methyl Macro-Prep resin containing dual functional groups (COO^−^ and butyl), achieving a final purity of 4.5 [87]. However, the recovery yield was not reported. It is also noteworthy that this approach required an additional purification stage, as the authors processed a previously purified fraction obtained by ammonium sulfate precipitation, which exhibited an initial purity of 0.86 [87]. More recently, Chen et al. (2018) employed a phenyl-based hydrophobic resin to purify a Spirulina crude extract rich in PC [88]. Unlike the fixed-bed chromatographic approach implemented in the present work, their system operated as a stirred fluidized bed, achieving a high PC recovery yield of 90.4% [88]. Although this configuration enabled substantial sample concentration, the resulting PC purity (1.56) remained well below the analytical-grade purity required for high-value applications and markedly lower than the purity levels achieved in the current study [88]. The work of Lauceri et al. (2019) represents the most successful effort reported to date, achieving analytical-grade PC recovery yields ranging from 75% to 82% [9]. However, their approach relies on hydrophobic membranes rather than a packed chromatographic column, which, despite offering advantages in processing speed and throughput, presents limitations in terms of loading capacity, resolution, operational flexibility, and cost per unit surface area [47].

In the present work, analytical-grade purity was achieved for PC but not for APC, an expected outcome given the inherently lower initial purity and extraction yield of APC in the Spirulina crude extract, which renders its purification to analytical grade more challenging. Nevertheless, the substantial enrichment achieved for APC remains relevant within the proposed biorefinery framework, since APC may still be valorized for applications that do not require analytical-grade purity, thereby contributing to a more comprehensive utilization of the cyanobacterial biomass. Despite this, when compared with previously reported APC purification studies, the present work yields a higher purification factor, underscoring the improved efficiency of the proposed process. For example, an increase in APC purity from 0.8 to 5.2—equivalent to a 6.3-fold improvement—was reported by Yan et al. (2011) [60]. In the case of PC, purity was raised from 2.1 to 5.6, corresponding to a purification factor of 2.64 [60]. Both values are clearly lower than the maximum purification factors of 10.57 for APC and 10.05 for PC achieved with the chromatographic procedure in the present study. A comparable trend is observed when examining the purification factors reported by Su et al. (2010) and Zhang and Chen (1999), whose values also fall below those obtained in the current work [59,89].

### 4.2. LCA

Regarding the LCA of the analytical-grade PC production process developed in this work, the purification stage was identified as the main environmental hotspot across all impact categories in both Scenarios A1 and A2 (Tables S2 and S3 and Figure 6), as previously mentioned in Section 3.4.1, *Scenarios A1 and A2*. This is largely attributable to the production of ammonium sulfate—the kosmotropic salt employed in HIC to promote hydrophobic interactions between PC and the resin—which accounted for 91–97% of the total purification stage impact in Scenario A1, based on the process contribution analysis of the background system modelled using the Ecoinvent 3.9.1 and Sphera LCA databases (Figure S4, Supplementary Material). Industrially, this salt is obtained by reacting gaseous NH_3_ with H_2_SO_4_, followed by concentration and crystallization, as described in patent U.S. Patent 3,607,136 A [90]. The synthesis of both H_2_SO_4_ and NH_3_ is energy-intensive and depends heavily on electricity and fossil fuels, thereby exerting significant burdens on multiple environmental compartments. Additionally, chromatographic purification requires substantial volumes of mobile phase, further increasing the overall environmental impact [40,41,42]. Accordingly, replacing ammonium sulfate with a more environmentally sustainable alternative offering comparable HIC performance represents a key improvement opportunity. Among the kosmotropic salts reported as viable substitutes—including sodium sulfate, sodium citrate, and ammonium tartrate [91,92,93]—sodium sulfate was selected in this work on account of its high ionic strength, sufficient solubility to perform the PC elution step from 1.25 to 0 M, and excellent protein stability [94,95]. However, this substitution was not experimentally validated in the present work but was incorporated into the LCA as a simulated improvement scenario; its suitability for HIC-based PC purification therefore warrants experimental confirmation in future studies. It should be noted that sodium sulfate showed a greater influence on ADP compared to ammonium sulfate (as evinced in Figure 7 and Figure S4), as its production in the Ecoinvent 3.9.1 database is modelled via extraction from mineral deposits and natural brines. Nevertheless, modern sodium sulfate production is increasingly based on recovery from industrial brines and wastewaters [96,97], which would reduce its associated environmental burdens. Regardless, in Scenario A2, the contribution of sodium sulfate production to the purification stage impact ranged from 46 to 76% across impact categories (Figure S4, Supplementary Material), compared to 91–97% attributed to ammonium sulfate production in Scenario A1.

Setting aside the cultivation stage—common to all evaluated scenarios—the PC extraction stage represented the next most significant contributor to the overall environmental burden. In Scenarios A1 and A2, UAE with an aqueous [Emim]^+^[EtSO_4_]^−^ solution was employed in place of the conventional extraction techniques used in Scenarios B and C, leading to reduced energy consumption and lower solvent requirements, consistent with previous findings in the literature [98,99,100]. In addition to its superior extraction efficiency relative to conventional buffers for PBP extraction [36], [Emim]^+^[EtSO_4_]^−^ presents notable environmental advantages over other ILs and organic solvents, including the lower toxicity of imidazolium cations compared to tetraalkylammonium or pyridinium ILs [36,101], the reduced toxicity of short-chain relative to long-chain imidazolium ILs [101], and the straightforward synthesis of ethyl sulfate–based ILs [102]. Nevertheless, its production remains environmentally challenging, as it involves hazardous reagents and organic solvents and is energy-intensive. Within the extraction stage, imidazolium production accounted for 39.5–67% of the total impact across most categories, reaching up to 96% in human toxicity potential (HTP), while dimethyl sulfate contributed 3–54% depending on the category (Figure S5). Accordingly, increasing IL recovery from 74.8 to 90 wt% was incorporated as a key improvement in Scenario A2.

The combined implementation of both improvements in Scenario A2 led to a marked reduction in the overall environmental footprint, with decreases ranging from 65.9% to 89.8% across all impact categories. This improvement is primarily driven by the substitution of ammonium sulfate with sodium sulfate, which dominated impact mitigation in the purification stage (82.7–91.4% reduction across most categories), while increased IL recovery provided an additional, albeit comparatively smaller, contribution. The only exception was abiotic depletion potential (ADP), which increased by 14.2%, consistent with the upstream inventory assumptions associated with sodium sulfate production in the background database—an aspect discussed further below. In the extraction stage, the transition from Scenario A1 to A2 yielded consistent reductions across all impact categories (32.0–60.9%), with the lowest reduction observed for cumulative energy demand (CED) and larger reductions in toxicity- and emission-related categories, reflecting the environmental benefits of reducing fresh IL consumption as a result of improving recovery efficiency. These results highlight the critical influence of salt selection on the environmental performance of the HIC step, suggesting that sustainability criteria should be incorporated alongside conventional physicochemical considerations in salt selection. Although experimentally unvalidated in the present work, these findings provide a valuable basis for future studies exploring alternative kosmotropic salts with lower environmental burdens. In parallel, increasing IL recovery from 74.8 to 90 wt% further contributed to the environmental improvement observed in Scenario A2 (Tables S3 and S4).

Regarding Scenario B, although it involves the largest number and variety of purification steps, it emerges as the most environmentally favorable option (Figure 7). This advantage is mainly attributed to the type of chromatography employed: IEC requires considerably less salt than HIC (Table S3), and the pre-concentration achieved during the preliminary purification allows higher PC load (8.67 mg·mL^−1^ versus 1.55 mg·mL^−1^ in Scenarios A1 and A2) while removing competing proteins, thereby reducing the volume of mobile phase required (Table S4). In contrast, Scenario C eliminates a chromatographic step to achieve analytical-grade PC. Still, it is less environmentally favorable due to higher salt and PEG quantities needed, as well as the higher electricity demand required for ATPS phase separation via centrifugation (Table S2). This was reflected in the high cumulative energy demand (CED) associated with purification processes, including electricity consumption (329.16 MJ), PEG synthesis (893.90 MJ), and sodium phosphate production (186.33 MJ), which together accounted for a substantial share of the total system impacts.

From an operational point of view, Scenarios A1 and A2 offer notable advantages over Scenarios B and C by minimizing both the number and the variety of unit operations in the purification stage, which is reduced to a single step (Figures S1–S3). This simplification leads to reduced process complexity, shorter operational times, lower equipment requirements, and consequently fewer scalability constraints—factors that are critical for industrial implementation and for guiding future research. Moreover, the improvements implemented in Scenario A2 make it competitive with Scenario C, although it remains environmentally inferior to Scenario B according to the LCA results. These results highlight both the benefits and the limitations—some of them non-intuitive—of the evaluated scenarios, enabling direct comparison and supporting the development of more efficient and sustainable pathways for future analytical-grade PC production from cyanobacteria.

## 5. Conclusions

This study presents a streamlined and sustainability-oriented strategy for producing analytical-grade phycocyanin (PC) from Spirulina, combining ionic liquid (IL)–ultrasound-assisted extraction (UAE) with a single hydrophobic interaction chromatography (HIC) step.

First, the method demonstrated high efficiency for both phycocyanin (PC) and allophycocyanin (APC), achieving analytical-grade PC recovery rates of up to 50.4% using both fresh and reused IL. Spectroscopic analyses confirmed the preservation of native protein conformation and chromophore stability throughout extraction and purification.

Second, LCA results identified the purification stage as the dominant environmental hotspot, followed by cultivation and extraction. Targeted improvements—such as replacing ammonium sulfate with sodium sulfate and increasing IL recovery—led to a substantial overall reduction in environmental impacts, ranging from around 65% to 90% across most categories. The only exception was ADP, which showed a slight increase of 14.2%. These results underscore the importance of integrating sustainability criteria into chromatographic salt selection, an aspect often overlooked in downstream process design.

Third, an environmental comparison of the improved HIC process (Scenario A2) with the conventional IEC-based route (Scenario B) and the dual-ATPS route (Scenario C) showed that the proposed process achieves an intermediate environmental performance between these alternatives. Moreover, it offers clear operational advantages: achieving purification in a single chromatographic step reduces processing time, equipment requirements, and scale-up constraints—key considerations for commercial implementation. Despite its greater operational complexity, Scenario B remains the environmentally preferable option, largely because IEC requires less salt than HIC.

Overall, this work successfully develops an integrated and effective extraction–purification route for producing analytical-grade PC, identifies concrete opportunities for process improvement, and clarifies key trade-offs between process and environmental performance. By revealing both the strengths and the non-intuitive limitations of the evaluated pathways, it provides a robust basis for comparison. It supports the development of more efficient, scalable, and sustainable bioprocesses for future high-purity phycobiliprotein production from cyanobacteria.

## 6. Patents

As a result of the research, the authors have been granted a Spanish Patent (ES-3017658-B2).

## Figures and Tables

**Table 1:** PC and APC extracted yield (E_PC_, E_APC_) and PC and APC purity (P_PC_, P_APC_) of Spirulina crude extracts. Errors are expressed as ± standard deviation of the mean (*n* = 3).

Solvent	P_PC_	E_PC_ (mg g^−1^)	P_APC_	E_APC_ (mg·g^−1^)
IL aqueous solution (fresh IL)	0.57 ± 0.01	87.6 ± 1.7	0.27 ± 0.01	30.2 ± 1.4
IL aqueous solution (reused IL)	0.41 ± 0.01	84.5 ± 0.9	0.20 ± 0.01	29.3 ± 0.3

## Data Availability

The raw data supporting the conclusions of this article will be made available by the authors on request.

## Supplementary Materials

The following supporting information can be downloaded at: https://www.mdpi.com/article/10.3390/microorganisms14061328/s1, Table S1. Key literature-based considerations incorporated into the design of Scenarios B and C. Table S2. Life Cycle Inventory data for the proposed scenarios. Table S3. Reuse of the mobile phase in the chromatographic process for scenarios A1, A2, and B. Table S4. PC mass balance throughout the biorefinery stages: literature data and simulated results. Table S5. Experimental purity and recovery yield of PC and APC purifications using fresh and reused ILs.. Errors are expressed as ± standard deviation of the mean (n = 3). Different letters indicate statistically significant differences among groups (purification levels) based on post-hoc multiple comparison tests with adjustment for multiple comparisons (*p* < 0.05). Figure S1. Flowsheet diagram of scenarios A1 and A2. Figure S2. Flowsheet diagram of scenario B. Figure S3. Flowsheet diagram of scenario C. Figure S4. Normalized contributions of raw material and energy supply processes associated with the purification stage across the evaluated impact categories for the studied cases (A1, A2, B, and C). Ammonium sulfate (red); sodium phosphate (dark grey); deionized water (blue); electricity (yellow); PEG (purple); and sodium acetate (light grey). Figure S5. Normalized contributions of raw material and energy supply processes associated with the extraction stage across the evaluated impact categories for the studied cases (A1, A2, B, and C). Imidazole (dark blue); dimethyl sulfate (green); sodium phosphate (dark grey); deionized water (blue); electricity (yellow); and sodium acetate (light grey).

## Abbreviations

The following abbreviations are used in this manuscript:

AD	Anaerobic digestion
ADP	Abiotic depletion potential
AP	Acidification potential
APC	Allophycocyanin
ATPS	Aqueous two-phase system
BV	Bed volume
C	Cultivation stage
CED	Cumulative energy demand
E_PC_	Phycocyanin extraction stage
E_APC_	Extracted allophycocyanin
EP	Eutrophication potential
E_PC_	Extracted phycocyanin
FAETP	Freshwater aquatic ecotoxicity
FT	Freeze and thaw
GWP 100	100-year global warming potential
H&D	Harvesting and drying stage
HIC	Hydrophobic interaction chromatography
HTP	Human toxicity potential
IEC	Ionic exchange chromatography
IL	Ionic liquid
LCA	Life cycle assessment
LCI	Life cycle inventory
OD	Optical density
P	Phycocyanin purification stage
P_APC_	Purity of allophycocyanin
PC	Phycocyanin
PEG	Polyethylene glycol
PF	Purification factor
POCP	Photochemical ozone creation potential
P-p	Phycocyanin post-processing stage
P_PC_	Purity of phycocyanin
TETP	Terrestrial ecotoxicity potential
UAE	Ultrasound-assisted extraction
UF	Ultrafiltration
V	Spent biomass valorization

## References

[1] Costa J.A.V., Freitas B.C.B., Rosa G.M., Moraes L., Morais M.G., Mitchell B.G. (2019). Operational and economic aspects of *Spirulina*-based biorefinery. Bioresour. Technol..

[2] Braud L., McDonnell K., Murphy F. (2025). Beyond phycocyanin: Environmental life cycle assessment of a European pilot scale *Spirulina* biorefinery. Bioresour. Technol. Rep..

[3] Thevarajah B., Nishshanka G.K.S.H., Premaratne M., Nimarshana P.H.V., Nagarajan D., Chang J.-S., Ariyadasa T.U. (2022). Large-scale production of *Spirulina*-based proteins and c-phycocyanin: A biorefinery approach. Biochem. Eng. J..

[4] Ashaolu T.J., Samborska K., Lee C.C., Tomas M., Capanoglu E., Tarhan Ö., Taze B., Jafari S.M. (2021). Phycocyanin, a super functional ingredient from algae: Properties, purification, characterization, and applications. Int. J. Biol. Macromol..

[5] Fernández-Rojas B., Hernández-Juárez J., Pedraza-Chaverri J. (2014). Nutraceutical properties of phycocyanin. J. Funct. Foods.

[6] Moraes C.C., Kalil S.J. (2009). Strategy for a protein purification design using C-phycocyanin extract. Bioresour. Technol..

[7] de Amarante M.C.A., Braga A.R.C., Sala L., Moraes C.C., Kalil S.J. (2020). Design strategies for C-phycocyanin purification: Process influence on purity grade. Sep. Purif. Technol..

[8] Chaiklahan R., Chirasuwan N., Loha V., Tia S., Bunnag B. (2011). Separation and purification of phycocyanin from *Spirulina* sp. using a membrane process. Bioresour. Technol..

[9] Lauceri R., Zittelli G.C., Torzillo G. (2019). A simple method for rapid purification of phycobiliproteins from *Arthrospira platensis* and *Porphyridium cruentum* biomass. Algal Res..

[10] Tang Z., Zhao J., Ju B., Li W., Wen S., Pu Y., Qin S. (2016). One-step chromatographic procedure for purification of B-phycoerythrin from *Porphyridium cruentum*. Protein Expr. Purif..

[11] Mogany T., Kumari S., Swalaha F.M., Bux F. (2019). Extraction and characterisation of analytical grade C-phycocyanin from *Euhalothece* sp.. J. Appl. Phycol..

[12] Roy D., Pabbi S. (2022). A simple improved protocol for purification of C-phycocyanin from overproducing cyanobacteria and its characterization. J. Appl. Phycol..

[13] Patil G., Chethana S., Sridevi A.S., Raghavarao K.S.M.S. (2006). Method to obtain C-phycocyanin of high purity. J. Chromatogr. A.

[14] Zang F., Qin S., Ma C., Li W., Lin J. (2020). Preparation of high-purity R-phycoerythrin and R-phycocyanin from *Pyropia yezoensis* in membrane chromatography. J. Appl. Phycol..

[15] Lauceri R., Cavone C., Zittelli G.C., Kamburska L., Musazzi S., Torzillo G. (2023). High purity grade phycocyanin recovery by decoupling cell lysis from the pigment extraction: An innovative approach. Food Bioprocess Technol..

[16] Scorza L.C.T., Simon U., Wear M., Zouliatis A., Dimartino S., McCormick A.J. (2021). Evaluation of novel 3D-printed monolithic adsorbers against conventional chromatography columns for the purification of c-phycocyanin from *Spirulina*. Algal Res..

[17] O’Connor B.F., Cummins P.M., Hatti-Kaul R., Mattiasson B. (2017). Hydrophobic interaction chromatography. Isolation and Purification of Proteins.

[18] Jennissen H.P. (2016). Hydrophobic interaction chromatography. Encyclopedia of Life Sciences.

[19] McCue J.T. (2009). Theory and use of hydrophobic interaction chromatography in protein purification applications. Methods Enzymol..

[20] Ebrahimi A., Pazuki G., Mozaffarian M., Ahsaie F.G., Abedini H. (2023). Separation and purification of C-phycocyanin from *Spirulina platensis* using aqueous two-phase systems based on triblock thermosensitive copolymers. Food Bioprocess Technol..

[21] Porav A.S., Bocăneală M., Fălămaş A., Bogdan D.F., Barbu-Tudoran L., Hegeduş A., Dragoş N. (2020). Sequential aqueous two-phase system for simultaneous purification of cyanobacterial phycobiliproteins. Bioresour. Technol..

[22] Patil G., Raghavarao K.S.M.S. (2007). Aqueous two phase extraction for purification of C-phycocyanin. Biochem. Eng. J..

[23] Arashiro L.T., Boto-Ordóñez M., Van Hulle S.W.H., Ferrer I., Garfí M., Rousseau D.P.L. (2020). Natural pigments from microalgae grown in industrial wastewater. Bioresour. Technol..

[24] Cruz J.D., Vasconcelos V. (2023). Legal aspects of microalgae in the European food sector. Foods.

[25] Posten C. (2009). Design principles of photo-bioreactors for cultivation of microalgae. Eng. Life Sci..

[26] Imamoglu E. (2024). Artificial intelligence and/or machine learning algorithms in microalgae bioprocesses. Bioengineering.

[27] Dębowski M., Kazimierowicz J., Zieliński M. (2025). Multi-sensing monitoring of the microalgae biomass cultivation systems for biofuels and added value products synthesis—Challenges and opportunities. Appl. Sci..

[28] Barros A.I., Gonçalves A.L., Simões M., Pires J.C.M. (2015). Harvesting techniques applied to microalgae: A review. Renew. Sustain. Energy Rev..

[29] Zhou S., Lin M., Zhang X., Zhan L., Li R., Wu Y. (2024). Study of life cycle assessment: Transforming microalgae to biofuel through hydrothermal liquefaction and upgrading in organic or aqueous medium. J. Clean. Prod..

[30] Maity S., Mallick N. (2023). Unraveling C-phycocyanin extraction by dark incubation from marine cyanobacterium *Leptolyngbya valderiana*. Sustain. Chem. Pharm..

[31] Kovaleski G., Kholany M., Dias L.M.S., Correia S.F.H., Ferreira R.A.S., Coutinho J.A.P., Ventura S.P.M. (2022). Extraction and purification of phycobiliproteins from algae and their applications. Front. Chem..

[32] Córdova A., Catalán S., Carrasco V., Farias F.O., Trentin J., López J., Salazar F., Mussagy C.U. (2025). Sustainable assessment of ultrasound-assisted extraction of anthocyanins with bio-based solvents for upgrading grape pomace Cabernet Sauvignon derived from a winemaking process. Ultrason. Sonochem..

[33] Guler B.A., Tepe U., Imamoglu E. (2024). Sustainable point of view: Life cycle analysis for green extraction technologies. ChemBioEng Rev..

[34] Alreshidi M.A., Yadav K.K., Gunasekaran S., Gacem A., Sambandam P., Subbiah G., Bhutto J.K., Palanivel S., Fallatah A.M., El-Khair M.A.A. (2025). A review on the evolution of ionic liquids: Sustainable synthesis, applications, and future prospects. Mater. Today Sustain..

[35] Martins M., Vieira F.A., Correia I., Ferreira R.A.S., Abreu H., Coutinho J.A.P., Ventura S.P.M. (2016). Recovery of phycobiliproteins from the red macroalga *Gracilaria* sp. using ionic liquid aqueous solutions. Green Chem..

[36] Piera A., Espada J.J., Morales V., Rodríguez R., Vicente G., Bautista L.F. (2024). Optimised phycoerythrin extraction method from *Porphyridium* sp. combining imidazolium-based ionic liquids. Heliyon.

[37] Antecka A., Szeląg R., Ledakowicz S. (2025). A novel two-step purification process for highly stable C-phycocyanin of analytical grade purity and its properties. Appl. Microbiol. Biotechnol..

[38] Patil G., Chethana S., Madhusudhan M.C., Raghavarao K.S.M.S. (2008). Fractionation and purification of the phycobiliproteins from *Spirulina platensis*. Bioresour. Technol..

[39] Fabre J.-F., Niangoran N.U.F., Gaignard C., Buso D., Mouloungui Z., Valentin R. (2022). Extraction, purification and stability of C-phycocyanin from *Arthrospira platensis*. Eur. Food Res. Technol..

[40] Jungbauer A., Walch N. (2015). Buffer recycling in downstream processing of biologics. Curr. Opin. Chem. Eng..

[41] Wellhoefer M., Sprinzl W., Hahn R., Jungbauer A. (2014). Continuous processing of recombinant proteins: Integration of refolding and purification using simulated moving bed size-exclusion chromatography with buffer recycling. J. Chromatogr. A.

[42] Isaksson M., Andersson N., Nilsson B. (2025). Improving the sustainability of biopharmaceutical downstream processing through buffer recycling. J. Chromatogr. A.

[43] Imura Y., Tagawa T., Miyamoto Y., Nonoyama S., Sumichika H., Fujino Y., Yamanouchi M., Miki H. (2021). Washing with alkaline solutions in protein A purification improves physicochemical properties of monoclonal antibodies. Sci. Rep..

[44] Grönberg A., Eriksson M., Ersoy M., Johansson H.J. (2011). A tool for increasing the lifetime of chromatography resins. MAbs.

[45] Zhao L., Peng Y., Gao J., Cai W. (2014). Bioprocess intensification: An aqueous two-phase process for the purification of C-phycocyanin from dry *Spirulina platensis*. Eur. Food Res. Technol..

[46] Zeng W., Luo J., Grimi N. (2025). Maximizing C-phycocyanin purification efficiency from *Spirulina*: A synergistic strategy combining CaCl_2_ precipitation and membrane diafiltration. Sep. Purif. Technol..

[47] Boi C., Malavasi A., Carbonell R.G., Gilleskie G. (2020). A direct comparison between membrane adsorber and packed column chromatography performance. J. Chromatogr. A.

[48] Sánchez-Laso J., Piera A., Vicente G., Bautista L.F., Rodríguez R., Espada J.J. (2021). A successful method for phycocyanin extraction from *Arthrospira platensis* using [Emim][EtSO_4_] ionic liquid. Biofuels Bioprod. Biorefin..

[49] Sánchez-Laso J., Espada J.J., Rodríguez R., Vicente G., Bautista L.F. (2023). Novel biorefinery approach for phycocyanin extraction and purification and biocrude production from *Arthrospira platensis*. Ind. Eng. Chem. Res..

[50] Piera A., Sainz-Urruela C., Espada J.J., Morales V., Vicente G., Bautista L.F. (2026). One-step purification process to obtain analytical grade phycoerythrin from *Porphyridium* sp. using hydrophobic interaction chromatography HIC. Algal Res..

[51] Bennett A., Bogorad L. (1973). Complementary chromatic adaptation in a filamentous blue-green alga. J. Cell Biol..

[52] García A., Longo E., Murillo M., Bermejo R. (2021). Using a B-phycoerythrin extract as a natural colorant: Application in milk-based products. Molecules.

[53] Román R.B., Alvárez-Pez J.M., Fernández F.G.A., Grima E.M. (2002). Recovery of pure B-phycoerythrin from the microalga *Porphyridium cruentum*. J. Biotechnol..

[54] Glazer A.N. (1994). Phycobiliproteins—A family of valuable, widely used fluorophores. J. Appl. Phycol..

[55] Bermejo R., Whitton N. (2014). Phycocyanins. Cyanobacteria.

[56] de Amarante M.C.A., Júnior L.C.S.C., Sala L., Kalil S.J. (2020). Analytical grade C-phycocyanin obtained by a single-step purification process. Process Biochem..

[57] Sivasankari S., Vinoth M., Ravindran D., Baskar K., Alqarawi A.A., Abd_Allah E.F. (2021). Efficacy of red light for enhanced cell disruption and fluorescence intensity of phycocyanin. Bioprocess Biosyst. Eng..

[58] Patel A., Mishra S., Pawar R., Ghosh P.K. (2005). Purification and characterization of C-phycocyanin from cyanobacterial species of marine and freshwater habitat. Protein Expr. Purif..

[59] Zhang Y.-M., Chen F. (1999). A simple method for efficient separation and purification of c-phycocyanin and allophycocyanin from *Spirulina platensis*. Biotechnol. Tech..

[60] Yan S.-G., Zhu L.-P., Su H.-N., Zhang X.-Y., Chen X.-L., Zhou B.-C., Zhang Y.-Z. (2011). Single-step chromatography for simultaneous purification of C-phycocyanin and allophycocyanin with high purity and recovery from *Spirulina* (*Arthrospira*) *platensis*. J. Appl. Phycol..

[61] Bischof K., Rautenberger R., Brey L., Pérez-Lloréns J. (2006). Physiological acclimation to gradients of solar irradiance within mats of the filamentous green macroalga *Chaetomorpha linum* from southern Spain. Mar. Ecol. Prog. Ser..

[62] Bischof K. (2002). Effects of solar UV-B radiation on canopy structure of *Ulva* communities from southern Spain. J. Exp. Bot..

[63] Hanrieder N., Kujawa A., Seychelles A.B., Blanco M., Carballo J., Wilbert S. (2024). Estimation of maximum photovoltaic cover ratios in greenhouses based on global irradiance data. Appl. Energy.

[64] Maraveas C. (2019). Environmental sustainability of greenhouse covering materials. Sustainability.

[65] Rizzoli M., Lutzu G.A., Usai L., Fais G., Dessì D., Soto-Ramirez R., Cosenza B., Concas A. (2025). Photoautotrophic batch cultivation of *Limnospira (Spirulina) platensis*: Optimizing biomass productivity and bioactive compound synthesis through salinity and pH modulation. Mar. Drugs.

[66] de Souza D.S., Valadão R.C., de Souza E.R.P., Barbosa M.I.M.J., de Mendonça H.V. (2022). Enhanced *Arthrospira platensis* biomass production combined with anaerobic cattle wastewater bioremediation. Bioenergy Res..

[67] Raoof B., Kaushik B.D., Prasanna R. (2006). Formulation of a low-cost medium for mass production of *Spirulina*. Biomass Bioenergy.

[68] Affan M.-A., Lee D.-W., Al-Harbi S.M., Kim H.-J., Abdulwassi N.I., Heo S.-J., Oh C., Park H.-S., Ma C.W., Lee H.-Y. (2015). Variation of *Spirulina maxima* biomass production in different depths of urea-used culture medium. Braz. J. Microbiol..

[69] Guidi F., Gojkovic Z., Venuleo M., Assunçao P.A.C.J., Portillo E. (2021). Long-term cultivation of a native *Arthrospira platensis (Spirulina*) strain in Pozo Izquierdo (Gran Canaria, Spain): Technical evidence for a viable production of food-grade biomass. Processes.

[70] Al hattab M. (2015). Microalgae harvesting methods for industrial production of biodiesel: Critical review and comparative analysis. J. Fundam. Renew. Energy Appl..

[71] Sinetova M.A., Kupriyanova E.V., Los D.A. (2024). *Spirulina/Arthrospira/Limnospira*—Three names of the single organism. Foods.

[72] Lao I.K.M., Edullantes B. (2025). Growth, productivity, and size structure of *Spirulina* strain under different salinity levels: Implications for cultivation optimization. Phycology.

[73] Nowak D., Jakubczyk E. (2020). The freeze-drying of foods—The characteristic of the process course and the effect of its parameters on the physical properties of food materials. Foods.

[74] Ronda A., Martín-Lara M.A., Osegueda O., Castillo V., Blázquez G. (2018). Scale-up of a packed bed column for wastewater treatment. Water Sci. Technol..

[75] Babu B.V., Gupta S. (2005). Modeling and simulation of fixed bed adsorption column: Effect of velocity variation. i-Manag. J. Future Eng. Technol..

[76] Fernandes R., Campos J., Serra M., Fidalgo J., Almeida H., Casas A., Toubarro D., Barros A.I.R.N.A. (2023). Exploring the benefits of phycocyanin: From *Spirulina* cultivation to its widespread applications. Pharmaceuticals.

[77] Zhou Y., Huang Z., Liu Y., Li B., Wen Z., Cao L. (2024). Stability and bioactivities evaluation of analytical grade C-phycocyanin during the storage of *Spirulina platensis* powder. J. Food Sci..

[78] Huijbregts M.A.J., Hellweg S., Frischknecht R., Hendriks H.W.M., Hungerbühler K., Hendriks A.J. (2010). Cumulative energy demand as predictor for the environmental burden of commodity production. Environ. Sci. Technol..

[79] Helbling E.W., Barbieri E.S., Sinha R.P., Villafañe V.E., Häder D.-P. (2004). Dynamics of potentially protective compounds in Rhodophyta species from Patagonia (Argentina) exposed to solar radiation. J. Photochem. Photobiol. B.

[80] Bryant D.A. (1982). Phycoerythrocyanin and phycoerythrin: Properties and occurrence in cyanobacteria. Microbiology.

[81] Singh O., Lee P.-Y., Matysiak S., Bermudez H. (2020). Dual mechanism of ionic liquid-induced protein unfolding. Phys. Chem. Chem. Phys..

[82] Bihari M., Russell T.P., Hoagland D.A. (2010). Dissolution and dissolved state of cytochrome c in a neat, hydrophilic ionic liquid. Biomacromolecules.

[83] Lee P.-Y., Singh O., Bermudez H., Matysiak S. (2022). Recovery of enzyme structure and activity following rehydration from ionic liquid. Phys. Chem. Chem. Phys..

[84] MacColl R. (1991). Fluorescence studies on R-phycoerythrin and C-phycoerythrin. J. Fluoresc..

[85] Li C., Wu H., Xiang W., Wu H., Wang N., Wu J., Li T. (2022). Comparison of production and fluorescence characteristics of phycoerythrin from three strains of *Porphyridium*. Foods.

[86] Abalde J., Betancourt L., Torres E., Cid A., Barwell C. (1998). Purification and characterization of phycocyanin from the marine cyanobacterium *Synechococcus* sp. IO9201. Plant Sci..

[87] Soni B., Trivedi U., Madamwar D. (2008). A novel method of single step hydrophobic interaction chromatography for the purification of phycocyanin from *Phormidium fragile* and its characterization for antioxidant property. Bioresour. Technol..

[88] Chen K.-H., Wang S.S.-S., Show P.-L., Lin G.-T., Chang Y.-K. (2018). A rapid and efficient technique for direct extraction of C-phycocyanin from highly turbid *Spirulina platensis* algae using hydrophobic interaction chromatography in stirred fluidized bed. Biochem. Eng. J..

[89] Su H.-N., Xie B.-B., Chen X.-L., Wang J.-X., Zhang X.-Y., Zhou B.-C., Zhang Y.-Z. (2010). Efficient separation and purification of allophycocyanin from *Spirulina* (*Arthrospira*) *platensis*. J. Appl. Phycol..

[90] Smiley R.A., Vernon J.A. (1971). Recovery of Ammonium Sulfate. U.S. Patent.

[91] Kempen T., Cadang L., Fan Y., Zhang K., Chen T., Wei B. (2025). Online native hydrophobic interaction chromatography-mass spectrometry of antibody-drug conjugates. MAbs.

[92] Xiu L., Valeja S.G., Alpert A.J., Jin S., Ge Y. (2014). Effective protein separation by coupling hydrophobic interaction and reverse phase chromatography for top-down proteomics. Anal. Chem..

[93] Freitas S.S., Santos J.A.L., Prazeres D.M.F. (2009). Plasmid purification by hydrophobic interaction chromatography using sodium citrate in the mobile phase. Sep. Purif. Technol..

[94] Kreusser J., Jirasek F., Hasse H. (2021). Influence of salts on the adsorption of lysozyme on a mixed-mode resin. Adsorpt. Sci. Technol..

[95] Koehnlein W., Kastenmueller E., Meier T., Treu T., Falkenstein R. (2024). The beneficial impact of kosmotropic salts on the resolution and selectivity of protein A chromatography. J. Chromatogr. A.

[96] More K.S., Maree J.P., Mahlangu M. (2025). Optimising salt recovery—Four-year operational insights into Na_2_SO_4_ recovery from saline waters using pipe freeze-crystallization. Water.

[97] Shen B., Zhao B., Du H., Ren Y., Tang J., Liu Y., Hua Q., Wang B. (2024). Influence of organic impurities on fractional crystallization of NaCl and Na_2_SO_4_ from high-salinity coal chemical wastewater: Thermodynamics and nucleation kinetics analysis. Molecules.

[98] Beaudor M., Vauchel P., Pradal D., Aljawish A., Phalip V. (2023). Comparing the efficiency of extracting antioxidant polyphenols from spent coffee grounds using an innovative ultrasound-assisted extraction equipment versus conventional method. Chem. Eng. Process..

[99] Gharibzahedi S.M.T., Altintas Z. (2022). Ultrasound-assisted alcoholic extraction of lesser mealworm larvae oil: Process optimization, physicochemical characteristics, and energy consumption. Antioxidants.

[100] Berrouane N.E.H., Attal F.-S., Benchabane A., Saghour I., Bitam A., Gachovska T., Amiali M. (2022). Freeze–thaw-, enzyme-, ultrasound- and pulsed electric field-assisted extractions of C-phycocyanin from *Spirulina platensis* dry biomass. J. Food Meas. Charact..

[101] Costa S.P.F., Pinto P.C.A.G., Lapa R.A.S., Saraiva M.L.M.F.S. (2015). Toxicity assessment of ionic liquids with *Vibrio fischeri*: An alternative fully automated methodology. J. Hazard. Mater..

[102] Liu J., Li Z., Chen J., Xia C. (2009). Synthesis, properties and catalysis of novel methyl- or ethyl-sulfate-anion-based acidic ionic liquids. Catal. Commun..

