# Supplementary material for: High-Purity Phycocyanin Production from Cyanobacteria Using a Biorefinery Approach: Life Cycle Assessment and Comparative Process Benchmarking
Source: Microorganisms. 2026 Jun 13;14(6):1328. doi: 10.3390/microorganisms14061328 (PMC13304063; doi:10.3390/microorganisms14061328)
Supplement: Supplementary file 1 [file microorganisms-14-01328-s001.zip › microorganisms-4294043-supplementary.pdf]

SUPPLEMENTARY MATERIAL

High-Purity Phycocyanin Production from Cyanobacteria using a Biorefinery Approach: Life Cycle Assessment and Comparative Process Benchmarking

Alejandro Piera [1], Victoria Morales [1], Gemma Vicente [2,3], Luis Fernando Bautista [1,3] and Juan José Espada [2,*]

[1] Department of Chemical and Environmental Technology. ESCET, Universidad Rey Juan Carlos. 28933 Móstoles, Madrid, Spain; alejandro.piera@urjc.es (A.P.); victoria.morales@urjc.es (V.M.); fenando.bautista@urjc.es (LF.B.)

[2] Department of Chemical, Energy and Mechanical Technology. ESCET, Universidad Rey Juan Carlos. 28933 Móstoles, Madrid, Spain; gemma.vicente@urjc.es (G.V.); juanjose.espada@urjc.es (JJ.E.)

[3] Instituto de Investigación de Tecnologías para la Sostenibilidad, Universidad Rey Juan Carlos. 28933 Móstoles, Madrid, Spain; gemma.vicente@urjc.es (G.V.); fernando.bautista@urjc.es (LF.B.)

* Correspondence: juanjose.espada@urjc.es (JJ.E.); Tel.: +34 914888087

* Corresponding author: Juan José Espada Sanjurjo (JJE) Phone: +34 914888087

E-mail: juanjose.espada@urjc.es

Table S1. Key literature-based considerations incorporated into the design of Scenarios B and C.

				Operation set-up	Scenario B	Scenario C
Biorefinery stage	Cultivation			[Spirulina] (g·L^{-1})	0.48	
				Retention time (d)	8 [1,2]	
				temperature (º C)	25 [2–4]	
				growth medium	Zarrouk [1–3,5,6]	
	PC extraction			technique	FT (×3) [1]	
				solvent	Na- acetate buffer, 20 mM, pH 5.0 [1]	Na-phosphate buffer, 20mM, pH 7 [2,7,8]
				biomass:solvent ratio (mg·mL^{-1})	10 [1,3,9]	
				[PC] (mg·mL^{-1})	1.8 [1]	1.55
				PC (% w/w)	100 [1]	100 [1]
				P_{PC} (A620 nm/A280 nm)	1.0 [1–3]	1.0 [1–3]
	PC purification	Preliminary purification	ammonium sulfate–based fractional precipitation	saturation (%)	25 and 60 [1]	-
				time process	Overnight [6]	
				precipitated:diluent ratio (v/v)	0.52 [6]	
				[PC] (mg·mL^{-1})	-	
				PC (% w/w)	86.6 [1]	
				P_{PC} (A620 nm/A280 nm)	2.11 [1]	
			dialysis	inner:outer membrane volume ratio (v/v)	1:200 [5,10]	
				membrane	membrane-70, MWCO: 12–14 kDa [5,10]	
				time process	Overnight [5,10]	
				[PC] (mg·mL^{-1})	8.67	
				PC (% w/w)	80 [11]	
				P_{PC} (A620 nm/A280 nm)	2.745 [2,5]	

Table S1. (Continue)

IEC		stationary phase	Weak Base Anion [1–3,10,12]
		buffer solutions	50 mM NaCl, 20 mM Na-acetate buffer (5.0-3.6 pH) [1]
		total volume (mL)	200 [1,10,12]
	mobile phase	equilibration	Na-acetate buffer, NaCl 50 mM (40 mL)
		wash	Na-acetate buffer, NaCl 50 mM (60 mL) [1]
		gradient	50 mM NaCl, 20 mM Na-acetate buffer (5.0-3.6 pH) (100 mL) [1]
		regeneration	NaCl 2 M [13,14]
		BV (mL)	8 [2,6]
		height (cm)	3.98 [1]
		section (cm^2)	2.01 [1]
		sample load (BV)	1.25 [4,10]
		Q ($mL \cdot min^{-1}$)	1 [1,2]
		μ ($cm \cdot min^{-1}$)	0.497 [1]
		[PC] ($mg \cdot mL^{-1}$)	3.0
		PC (% w/w)	67.0 [1]
		P_{PC} (A620 nm/A280 nm)	>4 [1–6,10]

Table S1. (Continue)

ATPS	system	PEG/ K-phosphate buffer [7,8,15]
	ATPS cycles	2 [7,8,15]
	[PEG] (% w/w)	29.99 [8]
	[salt] (% w/w)	8.01 [8]
	tie line length	46.31, pH 6, 35º C [8]
	sample load (% w/w)	10 [8]
	phase volume ratio (v/v)	0.34 [8]
	separation	Centrifugation [7,8]
	time process (h)	24 [8]
	polymer recovery rate (% w/w)	78 [8]
	[PC] ($mg{\cdot}mL^{-1}$)	0.1 [8]
	PC (% w/w)	80.15 [8,15]
	P_{PC} (A620 nm/A280 nm)	3.92 [8]
UF	MWCO (kDa)	30 [8,15]
	[PC] ($mg{\cdot}mL^{-1}$)	0.33
	PC (% w/w)	72.15
	P_{PC} (A620 nm/A280 nm)	>4 [8,15]

Table S2. Life Cycle Inventory data for the proposed scenarios.

Stage		Material/energy		Unit	Scenarios A1	A2	B	C
C	Inputs	Growth medium (simplified Zarrouk´s medium)	$NaHCO_3$	$Kg·h^{-1}$	0.48		0.32	0.28
			$NaNO_3$	$Kg·h^{-1}$	0.14		0.09	0.08
			Water	$Kg·h^{-1}$	28.06		18.69	16.5
		CO_2	CO_2	$Kg·h^{-1}$	0.48		0.32	0.28
		Energy	Electricity	MJ	310.51		206.82	182.62
		Culture	Culture	$L·h^{-1}$	574.13		382.41	337.66
H&D	Inputs	Culture	Culture	$Kg·h^{-1}$	574.13		382.41	337.66
		Energy	Electricity	MJ	12.05		9.75	9.15
	Outputs	Waste stream H&D	$NaHCO_3$	$Kg·h^{-1}$	0.48		0.32	0.28
			$NaNO_3$	$Kg·h^{-1}$	$6.00·10^{-5}$		$4.00·10^{-5}$	$3.00·10^{-5}$
			Water	$Kg·h^{-1}$	28.06		18.69	16.5
		Biomass	*A. platensis*	$Kg·h^{-1}$	0.28		0.19	0.17
E	Inputs	Biomass	*A.platensis*	$Kg·h^{-1}$	0.28		0.19	0.17
		Extraction solvent	CH_3COOH	$Kg·h^{-1}$			$5.94·10^{-3}$	-
			CH_3COONa	$Kg·h^{-1}$	-		$1.09·10^{-2}$	-
			Na_2HPO_4	$Kg·h^{-1}$			-	$5.07·10^{-3}$
			NaH_2PO_4	$Kg·h^{-1}$			-	$6.88·10^{-3}$
			Water	$Kg·h^{-1}$	3.24	0.64	10.51	9.29
			IL	$Kg·h^{-1}$	0.85	0.34	-	-
		Energy	Electricity	MJ	16.57		23.99	104.48
	Outputs	Spent biomass	Cell debires	$Kg·h^{-1}$	1.84		1.05	0.82
		PC crude extract		$L·h^{-1}$	14.32		9.69	8.67
P	Inputs	PC crude extract		$L·h^{-1}$	14.32		9.69	8.67
		Mobile phase / ATPS chemicals	PEG	$Kg·h^{-1}$	-		-	11.11
			Na_2HPO_4	$Kg·h^{-1}$	1.73		-	3.07
			NaH_2PO_4	$Kg·h^{-1}$	2.34		-	4.16
			Wat·10r	$Kg·h^{-1}$	$3.25·10^3$		45.4	67.97
			$(NH_4)_2SO_4$	$Kg·h^{-1}$	214.42	-	3.51	-
			Na_2SO_4	$Kg·h^{-1}$	-	214.42	-	-
			CH_3COOH	$Kg·h^{-1}$			0.02	-
			CH_3COONa	$Kg·h^{-1}$	-		0.04	-
			NaCl	$Kg·h^{-1}$			0.97	-
		Energy	Electricity	MJ	0.11		29.05	118.92

Table S2. (Continue)

	Outputs	Waste stream P (salts)	Cell debris	$Kg{\cdot}h^{-1}$	$2.60{\cdot}10^{-3}$		$1.70{\cdot}10^{-3}$	-
			Na_2HPO_4	$Kg{\cdot}h^{-1}$	0.31		-	-
			NaH_2PO_4	$Kg{\cdot}h^{-1}$	0.41		-	-
			Water	$Kg{\cdot}h^{-1}$	$1.12{\cdot}10^{3}$		51.51	-
			$(NH_4)_2SO_4$	$Kg{\cdot}h^{-1}$	228.22	-	3.51	-
			Na_2SO_4	$Kg{\cdot}h^{-1}$		228.22		-
			CH_3COOH	$Kg{\cdot}h^{-1}$			$2.16{\cdot}10^{-2}$	-
			CH_3COONa	$Kg{\cdot}h^{-1}$	-		$3.98{\cdot}10^{-2}$	-
			NaCl	$Kg{\cdot}h^{-1}$			0.97	-
			Proteins	$Kg{\cdot}h^{-1}$	-	-	$6.43{\cdot}10^{-2}$	-
		Waste stream P (IL)	Water	$Kg{\cdot}h^{-1}$	2.82	1.12	-	46.87
			IL	$Kg{\cdot}h^{-1}$	0.85	0.34	-	-
			P·10G	$Kg{\cdot}h^{-1}$	-		-	11.11
			Na_2HPO_4	$Kg{\cdot}h^{-1}$	-	-	-	0.34
			NaH_2PO_4	$Kg{\cdot}h^{-1}$	-	-	-	0.46
			Proteins	$Kg{\cdot}h^{-1}$	-	-	-	0.06
			Cell debries	$Kg{\cdot}h^{-1}$	-	-	-	0
		Purified PC		$L{\cdot}h^{-1}$	328.14		3.5	33.48
P-p	Inputs	Purified PC		$L{\cdot}h^{-1}$	328.14		3.5	33.48
		P-p medium/solvent	Na_2HPO_4	$Kg{\cdot}h^{-1}$	0.0291		-	0.11
			NaH_2PO_4	$Kg{\cdot}h^{-1}$	0.0395		-	0.15
			Water	$Kg{\cdot}h^{-1}$	53.38		15.97	203.22
			CH_3COOH	$Kg{\cdot}h^{-1}$	-		0.01	-
			CH_3COONa	$Kg{\cdot}h^{-1}$			0.02	-
		Energy	Electricity	MJ	13.33		0.58	8.17
	Outputs	PC commercial product	Purified PC ([PC] >1 mg/mL)	$Kg{\cdot}h^{-1}$	0.01		0.01	0.01
		Waste stream P-p	NaCl	$Kg{\cdot}h^{-1}$	-	-	0.01	
			Na_2HPO_4	$Kg{\cdot}h^{-1}$	0.2		-	2.84
			NaH_2PO_4	$Kg{\cdot}h^{-1}$	0.27		-	3.85
			$(NH_4)_2SO_4$	$Kg{\cdot}h^{-1}$	0.21	-	-	-
			Na_2SO_4	$Kg{\cdot}h^{-1}$	-	0.21	-	-
			Water	$Kg{\cdot}h^{-1}$	369.58		16.14	223.08
			CH_3COOH	$Kg{\cdot}h^{-1}$	-		$9.13{\cdot}10^{-3}$	-
			CH_3COONa	$Kg{\cdot}h^{-1}$			$1.68{\cdot}10^{-2}$	-

Table S2. (Continue)

V	Inputs	Spent biomass	Wet cell debris	Kg·h^{-1}	1.84	1.05	0.82
		Diluent	Water	Kg·h^{-1}	17.28	11.68	10.4
		Inoculum	Anaerobic sludge	Kg·h^{-1}	36.26	24.15	21.32
		Co-substrate	Glycerol	Kg·h^{-1}	$2.90 \cdot 10^{-2}$	$2.00 \cdot 10^{-2}$	$1.70 \cdot 10^{-2}$
		Energy	Electricity	MJ	4.22	2.8	2.47
	Outputs	Biogas		Kg·h^{-1}	0.16	0.11	0.1
		Digestate		L·h^{-1}	148.26	98.75	87.19

* C: Cultivation; H&D: Harvesting and Drying; E: Extraction; P: Purification; P-p: Post-processing; V: Spent biomass valorization.

Table S3. Reuse of the mobile phase in the chromatographic process for scenarios A1, A2, and B.

Scenarios A1 and A2					
	reused mobile phase (L)	Na_2HPO_4 (kg)	NaH_2PO_4 (kg)	$(NH_4)_2SO_4$ / Na_2SO_4 kg)	water (kg)
elution buffer b	198.07	0.11	0.15	0.00	196.89
elution buffer a	594.20	0.38	0.51	228.42	462.14
column regeneration	792.27	0.00	0.00	0.00	788.08
**Scenario B**					
	reused mobile phase (L)	CH_3COOH (kg)	CH_3COONa (kg)	NaCl (kg)	water (kg)
elution buffer	3.89	$2.19 \cdot 10^{-3}$	$4.02 \cdot 10^{-3}$	$1.13 \cdot 10^{-2}$	3.86
column regeneration	3.89	0	0	0.45	3.64

Table S4. PC mass balance throughout the biorefinery stages: literature data and simulated results.

	Scenarios A1 and A2					
	PC (% w/w)		PC ($mg\cdot mL^{-1}$)		P_{PC} (A620/A280)	
	experimental	simulated	experimental	simulated	experimental	simulated
Spirulina crude extract	100.00	98.98	1.55	1.63	0.57	-
HIC	50.44	49.92	0.04	0.04	>4	>4
UF 1	-	-	-	0.09	>4	>4
UF 2	-	-	-	0.45	>4	>4
diafiltration	-	42.44	-	1.01	>4	>4
	Scenario B					
	PC (% w/w)		PC ($mg\cdot mL^{-1}$)		P_{PC} (A620/A280)	
	literature	simulated	literature	simulated	literature	simulated
Spirulina crude extract	100.00	99.04	-	1.60	0.97	-
precipitation 1	-	99.04	-	1.51	-	-
precipitation 2	86.59	80.10	-	8.61	2.11	-
diafiltration	80.10	80.10	-	8.67	2.75	-
IEC	67.04	67.11	-	3.01	5.59	>4
diafiltration	-	63.73	-	3.03	-	>4
	Scenario C					
	PC (% w/w)		PC ($mg\cdot mL^{-1}$)		P_{PC} (A620/A280)	
	literature	simulated	literature	simulated	literature	simulated
Spirulina crude extract	100.00	98.99				
ATPS 1	90.00	89.11	0.11	0.37	3.92	
ATPS 2	-	80.16		0.33	-	>4
UF + Diafiltration	-	72.15		1.01	5.90	>4

Table S5. Experimental purity and recovery yield of PC and APC purifications using fresh and reused ILs.. Errors are expressed as ± standard deviation of the mean (n = 3). Different letters indicate statistically significant differences among groups (purification levels) based on post-hoc multiple comparison tests with adjustment for multiple comparisons ($p < 0.05$).

	Purification Level	Purity	Yield (%)
PC purification using fresh IL	L1	2.21 ± 0.37^{a}	89.42 ± 1.58^{A}
	L2	3.36 ± 0.19^{b}	85.28 ± 2.05^{A}
	L3	$3.64 \pm 0.16^{b,c}$	76.87 ± 2.38^{B}
	L4	4.04 ± 0.14^{c}	50.44 ± 1.99^{C}
	L5	4.17 ± 0.04^{c}	30.40 ± 2.08^{D}
PC purification using reused IL	L1	1.88 ± 0.36^{a}	91.92 ± 1.51^{A}
	L2	3.04 ± 0.28^{b}	86.88 ± 2.24^{B}
	L3	3.66 ± 0.25^{c}	73.35 ± 1.16^{C}
	L4	$4.04 \pm 0.07^{c,d}$	47.83 ± 1.91^{D}
	L5	4.12 ± 0.14^{d}	37.39 ± 0.08^{E}
APC purification using fresh IL	L1	1.05 ± 0.22^{a}	76.17 ± 1.84^{A}
	L2	1.55 ± 0.09^{b}	63.54 ± 1.90^{B}
	L3	1.72 ± 0.06^{b}	55.66 ± 1.88^{C}
	L4	1.76 ± 0.05^{b}	46.79 ± 2.02^{D}
	L5	1.81 ± 0.06^{b}	16.93 ± 1.35^{E}
APC purification using reused IL	L1	1.06 ± 0.18^{a}	71.53 ± 2.10^{A}
	L2	1.50 ± 0.19^{b}	64.08 ± 2.58^{B}
	L3	1.77 ± 0.15^{c}	58.07 ± 1.72^{C}
	L4	$1.90 \pm 0.11^{c,d}$	45.26 ± 2.23^{D}
	L5	2.11 ± 0.01^{d}	19.19 ± 0.15^{E}

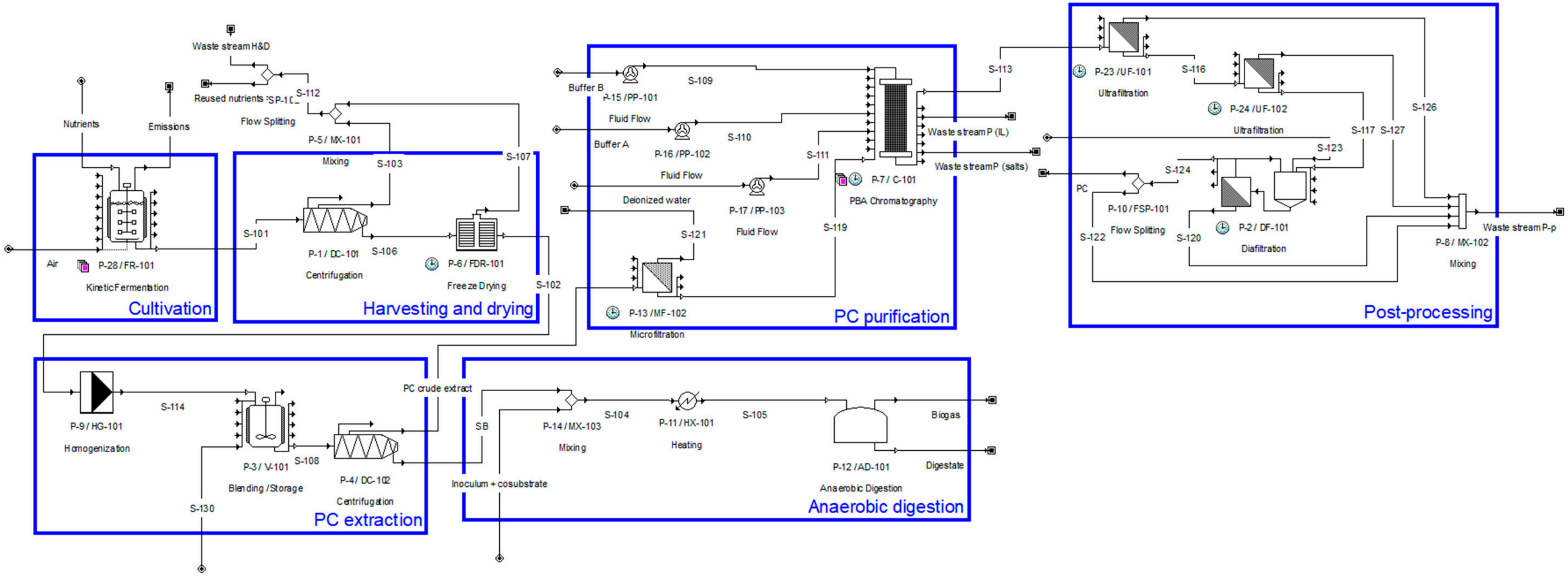

Figure S1. Flowsheet diagram of scenarios A1 and A2.

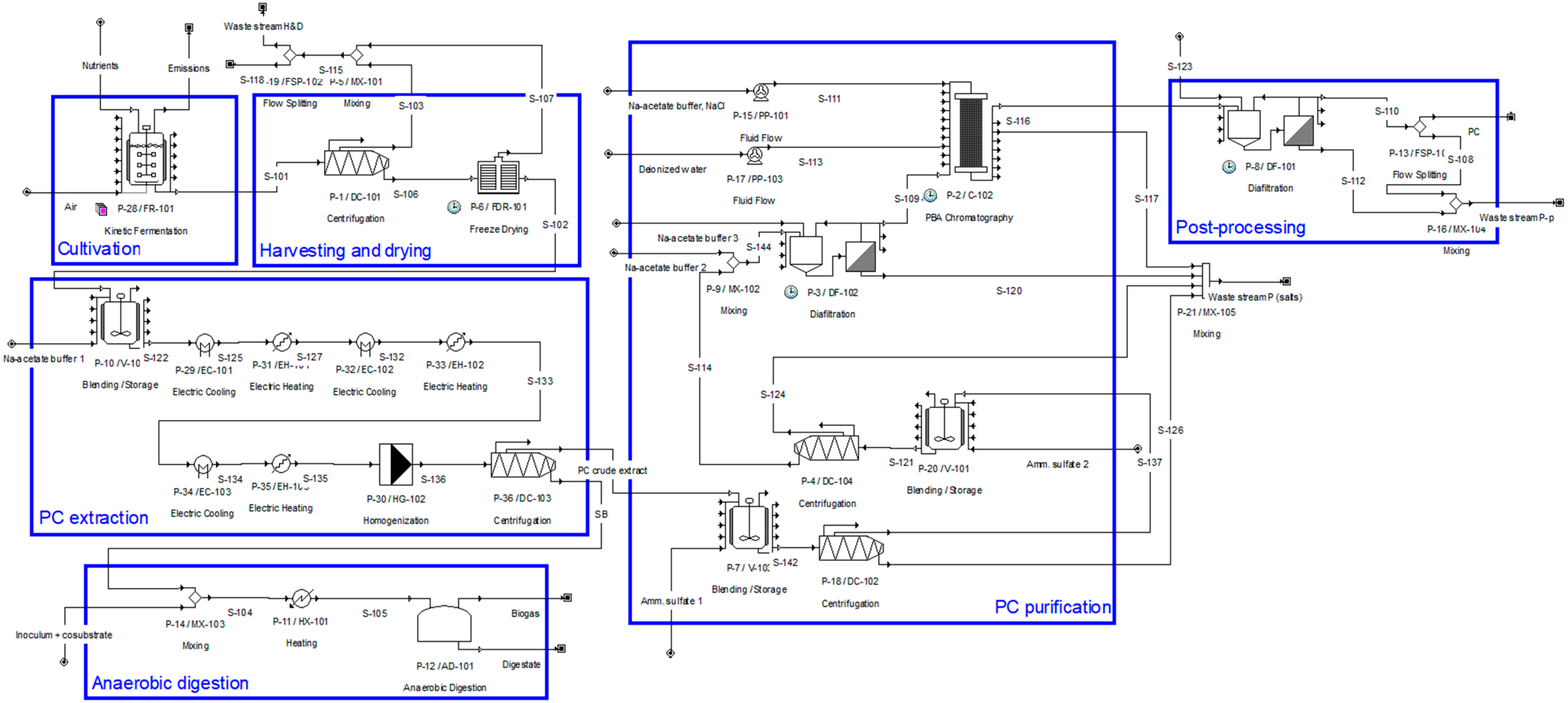

Figure S2. Flowsheet diagram of scenario B.

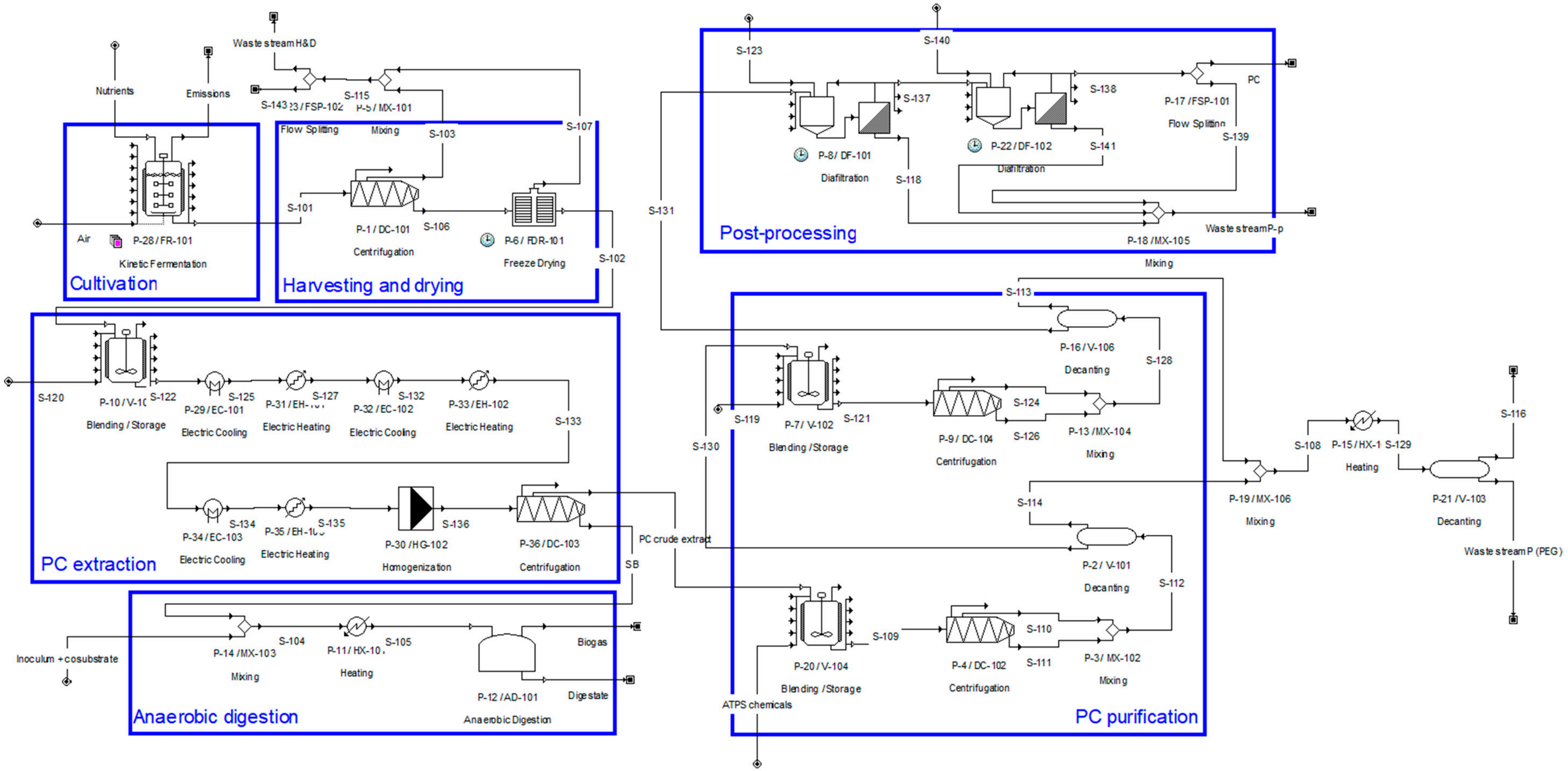

Figure S3. Flowsheet diagram of scenario C.

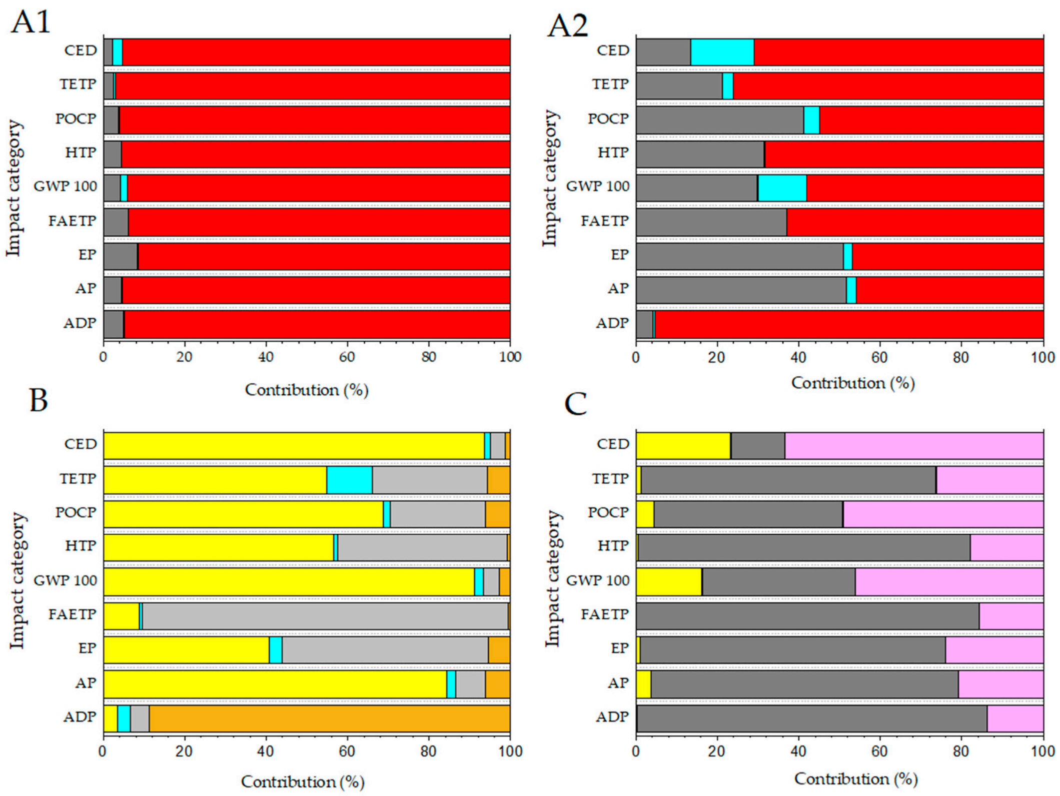

Figure S4. Normalized contributions of raw material and energy supply processes associated with the purification stage across the evaluated impact categories for the studied cases (A1, A2, B, and C). Ammonium sulfate (red); sodium phosphate (dark grey); deionized water (blue); electricity (yellow); PEG (purple); and sodium acetate (light grey).

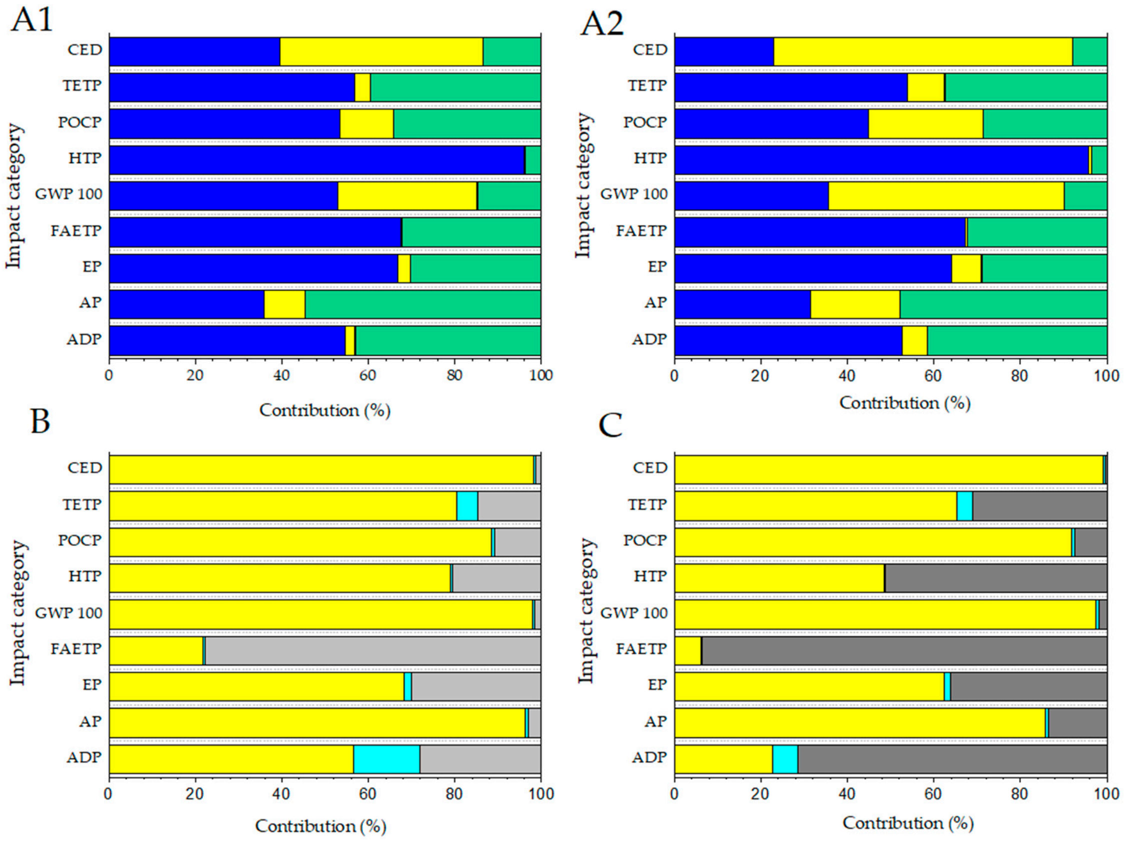

Figure S5. Normalized contributions of raw material and energy supply processes associated with the extraction stage across the evaluated impact categories for the studied cases (A1, A2, B, and C). Imidazole (dark blue); dimethyl sulfate (green); sodium phosphate (dark grey); deionized water (blue); electricity (yellow); and sodium acetate (light grey).

References

[1] S.-G. Yan, L.-P. Zhu, H.-N. Su, X.-Y. Zhang, X.-L. Chen, B.-C. Zhou, Y.-Z. Zhang, Single-step chromatography for simultaneous purification of C-phycocyanin and allophycocyanin with high purity and recovery from *Spirulina (Arthrospira) platensis, J. Appl. Phycol.* 23 (2011) 1–6. https://doi.org/10.1007/s10811-010-9525-7.

[2] Y.-M. Zhang, F. Chen, A simple method for efficient separation and purification of c-phycocyanin and allophycocyanin from *Spirulina platensis*, *Biotechnology Techniques* 13 (1999) 601–603. https://doi.org/10.1023/A:1008914405302.

[3] A. Patel, S. Mishra, R. Pawar, P.K. Ghosh, Purification and characterization of C-Phycocyanin from cyanobacterial species of marine and freshwater habitat, *Protein Expr. Purif.* 40 (2005) 248–255. https://doi.org/10.1016/j.pep.2004.10.028.

[4] M.C.A. de Amarante, L.C.S. Corrêa Júnior, L. Sala, S.J. Kalil, Analytical grade C-phycocyanin obtained by a single-step purification process, *Process Biochemistry* 90 (2020) 215–222. https://doi.org/10.1016/j.procbio.2019.11.020.

[5] S. Sivasankari, M. Vinoth, D. Ravindran, K. Baskar, A.A. Alqarawi, E.F. Abd_Allah, Efficacy of red light for enhanced cell disruption and fluorescence intensity of phycocyanin, *Bioprocess Biosyst. Eng.* 44 (2021) 141–150. https://doi.org/10.1007/s00449-020-02430-5.

[6] C.C. Moraes, S.J. Kalil, Strategy for a protein purification design using C-phycocyanin extract, *Bioresour. Technol.* 100 (2009) 5312–5317. https://doi.org/10.1016/j.biortech.2009.05.026.

[7] A.S. Porav, M. Bocăneală, A. Fălămaş, D.F. Bogdan, L. Barbu-Tudoran, A. Hegeduş, N. Dragoş, Sequential aqueous two-phase system for simultaneous purification of cyanobacterial phycobiliproteins, *Bioresour. Technol.* 315 (2020) 123794. https://doi.org/10.1016/j.biortech.2020.123794.

[8] A. Ebrahimi, G. Pazuki, M. Mozaffarian, F.G. Ahsaie, H. Abedini, Separation and purification of C-phycocyanin from *Spirulina platensis* using aqueous two-phase systems based on triblock thermosensitive copolymers, *Food Bioproc. Tech.* 16 (2023) 2582–2597. https://doi.org/10.1007/s11947-023-03057-6.

[9] J. Sánchez-Laso, A. Piera, G. Vicente, L.F. Bautista, R. Rodríguez, J.J. Espada, A successful method for phycocyanin extraction from *Arthrospira platensis* using [Emim] [EtSO4] ionic liquid, *Biofuels, Bioproducts and Biorefining* 15 (2021) 1638–1649. https://doi.org/10.1002/bbb.2275.

[10] D. Kumar, D.W. Dhar, S. Pabbi, N. Kumar, S. Walia, Extraction and purification of C-phycocyanin from *Spirulina platensis* (CCC540), *Indian J. Plant Physiol.* 19 (2014) 184–188. https://doi.org/10.1007/s40502-014-0094-7.

[11] J. Sánchez-Laso, J.J. Espada, R. Rodríguez, G. Vicente, L.F. Bautista, Novel biorefinery approach for phycocyanin extraction and purification and biocrude production from *Arthrospira platensis*, *Ind. Eng. Chem. Res.* 62 (2023) 5190–5198. https://doi.org/10.1021/acs.iecr.2c03683.

[12] X. Liao, B. Zhang, X. Wang, H. Yan, X. Zhang, Purification of C-phycocyanin from *Spirulina platensis* by single-step ion-exchange xhromatography, *Chromatographia* 73 (2011) 291–296. https://doi.org/10.1007/s10337-010-1874-5.

[13] S. Zheng, X. Li, X. Zhang, W. Wang, S. Yuan, Effect of inorganic regenerant properties on pharmaceutical adsorption and desorption performance on polymer anion exchange resin, *Chemosphere* 182 (2017) 325–331. https://doi.org/10.1016/j.chemosphere.2017.05.042.

[14] J.A. Korak, R. Huggins, M. Arias-Paic, Regeneration of pilot-scale ion exchange columns for hexavalent chromium removal, *Water Res.* 118 (2017) 141–151. https://doi.org/10.1016/j.watres.2017.03.018.

[15] G. Patil, K.S.M.S. Raghavarao, Aqueous two phase extraction for purification of C-phycocyanin, *Biochem. Eng. J.* 34 (2007) 156–164. https://doi.org/10.1016/j.bej.2006.11.026.
